# Time Constraints Modulate the Effects of Predator Cues and a Metal Across Life Stages in a Damselfly

**DOI:** 10.1111/eva.70169

**Published:** 2025-10-31

**Authors:** Nermeen R. Amer, Maria J. Golab, Robby Stoks, Guillaume Wos, Szymon Sniegula

**Affiliations:** ^1^ Institute of Nature Conservation, Polish Academy of Sciences Krakow Poland; ^2^ Department of Entomology, Faculty of Science Cairo University Giza Egypt; ^3^ Evolutionary Stress Ecology and Ecotoxicology, University of Leuven Leuven Belgium

**Keywords:** carry‐over effects, life history trade‐offs, metal, non‐consumptive effect, phenotypic plasticity, stressor interactions, toxicological effect

## Abstract

Animals are increasingly exposed to multiple co‐occurring stressors. Environmental factors such as seasonal time constraints (TC), predation risk, and pollutants strongly influence fitness‐related traits in aquatic organisms. Yet, the interactive effects of such stressors, especially across life stages, remain unclear. We examined immediate and delayed effects of predator cue exposure during the post‐overwintering egg stage and the larval stage, both subjected to early‐ or late‐season photoperiods, and how these factors interacted with subsequent larval exposure to predator cues and copper in the damselfly *Lestes sponsa*. Copper was used due to its known effects as a pesticide on aquatic invertebrates. We measured immediate effects of egg predator cue on egg hatching (development time), carry‐over effects on larval survival and growth rate, and behavioural (activity, resting, freezing, feeding) and physiological (oxidative damage, cellular energy allocation) traits after larval exposure to metal and predator cues. Several pairwise stressor interactions occurred, but none were modified by a third stressor. Predator cues during the egg stage delayed hatching under strong TC and led to sex‐specific carry‐over effects: males had reduced growth under strong TC. Copper increased oxidative damage only under weak TC, suggesting that strong TC can induce a hormetic antioxidant response. Short‐term copper exposure did not affect survival, behaviour, or net energy budget. However, predator exposure during the egg stage modified energy allocation, increasing it under weak TC and reducing it under strong TC, indicating context‐dependent trade‐offs. Behavioural responses were shaped by predator cues and TC; fast‐growing larvae under strong TC increased activity and feeding, while predator‐exposed individuals reduced these behaviours. These findings show how environmental stressors interact across life stages and traits, shaping plastic, sex‐specific responses. By integrating natural and anthropogenic stressors with life‐history timing, our study advances understanding of how ecological and evolutionary processes shape stress responses.

## 1 Introduction

Given organisms are increasingly facing multiple stressors worldwide, the focus in stress ecology has shifted from testing the effects of single stressors toward their combined effects (Orr et al. 2024). However, combined effects of stressors are often difficult to predict as effects may interact with each other (Simmons et al. 2021). Furthermore, in the context of global change, there is an increasing interest in studying the effects of factors directly related to human activities on organisms, and how these interact with biotic and abiotic stressors (Gissi et al. 2021). One of the most important factors linked with human activities is contamination with heavy metals, which are widely present in the natural environment, affecting individual organisms, populations, and communities (Paithankar et al. 2021). Since heavy metals can interact with widespread biotic and abiotic stressors, like biological invasions (Sornom et al. 2012) and temperature (Dinh Van et al. 2013; Hayden et al. 2015), it is important to study the combined effects of pollutants with biotic and abiotic stressors to assess the real impact of global change. Predation risk is well‐documented in its ability to amplify the effect of metals, with effects on prey behaviour (Amer et al. 2022; Bonnard et al. 2009), life history (Becker and Beckerman 2022; Rohr et al. 2006), and physiology (Boukadida et al. 2022; Frasco et al. 2005). For example, copper and predation risk had synergistic effects and considerably reduced the respiration rate of two copepod prey (Lode et al. 2020), and a combination of cadmium and lead synergistically reduced the feeding rate and the activity of isopods exposed to predator‐induced stress (Van Ginneken et al. 2018). Despite the fact that interactions are more likely with more stressors, the large majority of studies focused on two stressors, and much less considered three stressors (Diamant et al. 2023; Orr et al. 2024). Such an approach not only increases realism but also reveals that in the majority of cases, two‐stressor interactions may strongly depend on a third stressor (Diamant et al. 2023). To the best of our knowledge, studies that combined a pollutant with both biotic and abiotic stressors are missing from the literature.

One widespread stressor that remains understudied in multi‐stressor studies is the seasonal time constraint (TC)—a limitation on the period available for growth and development due to predictable environmental changes (Gotthard 2001). Seasonally time‐stressed ectotherms often face deadlines imposed by TC, for example, the arrival of suboptimal thermal conditions later in the season or, for semiaquatic organisms, the need to metamorphose before pond drying (Johansson et al. 2001; Lind et al. 2008). In response, ectotherms adjust their life history to changes in variables such as day length (photoperiod), which is the most reliable environmental cue in seasonal environments (Bradshaw and Holzapfel 2007). In response to cues indicating time constraints, animals typically accelerate their life history, which is associated with changes in behavioural (such as an increased food intake) and physiological traits (such as an increased metabolic rate) (Plaistow et al. 2005; Tüzün et al. 2020). Given time constraints are energetically costly, as speeding up biological processes raises metabolic rates and increases energy use (Stoks, De Block, and McPeek 2006), synergistic effects with other stressors are expected (Liess et al. 2016). Yet, also reduced responses to other stressors can be expected. Specifically, theory predicts that animals would prioritize an adaptive response to TC, such as fast growth and development, instead of a strong response to predation risk (Abrams et al. 1996; Rowe and Ludwig 1991; Werner and Anholt 1993), which indeed has been supported in empirical studies (Stoks, De Block, Slos, et al. 2006). For example, eggs oviposited by blue‐tailed damselfly (*Ischnura elegans*) females late in the season—therefore experiencing a shorter time window before the end of the growth season—compensated by shortening their development time, but only in the absence of predator cues (Sniegula et al. 2024). How time constraints affect sensitivity to pollutants remains largely unexplored. One rare study revealed that time constraints associated with a later egg hatching date unexpectedly reduced the impact of a pollutant (Tüzün and Stoks 2017). The above examples suggest that the interactive effects of stressors like TC, pollutants, and predation risk may not always be additive and these stressors may magnify or dampen each other's effects. Instead, under certain ecological scenarios, one stressor may mitigate the impact of another. This highlights the need to consider interactive and potentially compensatory effects among multiple stressors, rather than assuming uniformly negative outcomes.

Predation risk is a fundamental ecological factor that can strongly influence life history traits, behaviour, and physiology of organisms (Benard 2004; Clinchy et al. 2013). Exposure to predator cues often induces changes such as altered foraging strategies, decreased activity, and shifts in growth and development rates, which can reduce mortality by predation but also carry fitness and energetic costs (Stoks, De Block, Van de Meutter, and Johansson 2005; Wang, Tüzün, et al. 2022; Wos et al. 2025). These phenotypic responses to predation risk may occur across multiple developmental stages, from eggs to larvae and beyond, shaping individual performance and population dynamics (Amer et al. 2024; Touchon et al. 2006). Given its widespread and complex impact, predation risk requires investigation as a key stressor, not only because of its direct effects but also due to its potential to interact with other environmental stressors.

Another less explored topic in multiple stressor research is that stressor effects may not only have delayed impacts on the next life stage, but may also interact across life stages. The majority of ectotherms have a complex life cycle (Kingsolver et al. 2011; Stoks and Córdoba‐Aguilar 2012) and delayed effects across life stages, also called carry‐over effects, are widespread (Moore and Martin 2019; Salis et al. 2018). Several studies indeed reported carry‐over effects of pollutants such as metals in later life stages (Kimberly and Salice 2014; Tüzün and Stoks 2017). For example, while larval toads ( *Anaxyrus terrestris* ) exposed to copper and having parents from a contaminated wetland showed no effect in the larval stage, they demonstrated a carry‐over effect, resulting in reduced survival in the postmetamorphic stage (Rumrill et al. 2018). To a much lesser extent, it has been shown that delayed effects may interact with stressors in another life stage. For example, in the frog *Limnodynastes peronii* , the thermal environment experienced during embryonic development modulated the sublethal effects of the insecticide endosulfan on tadpole growth and predator avoidance (Broomhall 2004). Similarly, in damselflies, early life exposure to a heat wave altered the physiological response to subsequent pesticide exposure during the larval stage, with reduced adult fat content observed only in individuals not previously exposed to thermal stress (Sniegula, Janssens, and Stoks 2017). Hence, to have a better picture of the influence of stressors, it is important to track their interactive effects across developmental stages.

Here, we explored the single and combined effects of larval exposure to predator cues and to a metal (copper), and how these were modulated by time constraints and carry‐over effects by exposure to predator cues experienced in the egg stage. These effects were examined on a set of life history, behavioural, and physiological traits. Sex was recorded and included in all models, as males and females may differ in traits such as growth rate and energy allocation (Donelan and Trussell 2020). We used a strictly annual (i.e., univoltine) damselfly *Lestes sponsa* that experiences different degrees of time constraints, associated with natural variation in the egg hatching date. Time constraints were generated by rearing damselflies in either early (weak TC) or late (strong TC) season photoperiods (Johansson et al. 2001). As predator, we used the invasive alien spiny‐cheek crayfish *Faxonius limosus* that occupy the study area (Kouba et al. 2014). We chose the metal copper because it is widely used as an algicide and fungicide (Goswami et al. 2019; Lusk and Chapman 2020) and is an important threat to the diversity of aquatic insects (Brix et al. 2011; Reza and Ilmiawati 2020). We hypothesized that short‐term larval exposure to the predator cues and the metal would elevate metabolic activity under stress, though potentially at a cost to growth and survival (Nordberg et al. 2014; Slos and Stoks 2008). Time constraints were predicted to increase growth rate as a strategy to avoid a mass loss under the shorter development times (Dmitriew 2011; Johansson and Rowe 1999). In terms of behavioural responses, we hypothesize that predator cues and copper exposure would reduce larval activity and feeding traits because of risk avoidance and reduced energy and/or neurological effects, respectively (Amer et al. 2022; Reeves et al. 2011). Time constraints, in contrast, were predicted to increase behavioural activity to promote rapid growth (Johansson and Rowe 1999; Sniegula, Golab, and Johansson 2017). However, behavioural plasticity might be constrained when these stressors act in combination. We also hypothesized that copper and predator cues, alone and in combination, would elevate oxidative damage and reduce the net energy budget due to increased metabolic demands (Gaetke and Chow 2003; Janssens and Stoks 2013). Time constraints were expected to modulate these effects, potentially inducing hormetic responses that enhance antioxidant defences or energy reallocation because of the expected faster growth under TC (Chainy et al. 2016; Costantini et al. 2010). Finally, we predicted that early life exposure to predator cues (egg stage) would carry over to larval traits and intensify the impact of stressors experienced during the larval stage (stress accumulation) (Moore and Martin 2019; Sniegula et al. 2020). Across all response types, we further hypothesized that three‐way interactions among time constraints, predator cues, and metal exposure could emerge, reflecting the potential for complex, non‐additive effects that vary across traits and developmental stages (Becker and Beckerman 2022; Orr et al. 2024; Simmons et al. 2021).

## 2 Material and Methods

### 2.1 Study Species, Collection, and Rearing

*Lestes sponsa* is a common damselfly with widespread distribution, ranging from Western Europe across to East Asia, including Japan (Boudot and Raab 2015). It is an obligatory univoltine (one generation/year) damselfly (Corbet 1956). In central Europe, the flying season is between late spring and fall (Corbet 1956; Johansson et al. 2010). Adult females lay eggs endophytically into soft‐stemmed emergent and aquatic plants; oviposition typically begins at or just above the water surface and frequently continues underwater, so many eggs are submerged immediately after laying, while eggs placed above the waterline may later be flooded as water levels rise (Brooks and Cham 2014; Dolný et al. 2014). Individuals overwinter in diapausing eggs. Hatching occurs during the following spring, and the timing of hatching is temperature and photoperiod dependent (Corbet 1956; Sniegula et al. 2016). However, variation in hatching dates both within and between populations may be influenced by increasingly frequent droughts (Gebrechorkos et al. 2025; Hogreve and Suhling 2022). Post‐diapause eggs require rehydration to hatch (Sawchyn and Church 1973), and spring droughts can substantially delay this process. Sexes are easily distinguishable under the microscope at the final larval instars prior to emergence (based on copulatory organs). The damselfly exhibits substantial, often sex‐specific variation in hatching dates, larval growth rates, and emergence dates in nature and laboratory conditions (Johansson et al. 2010; Raczyński et al. 2021; Sniegula et al. 2014). Also, individuals that hatch later in the season experience stronger seasonal time constraints (strong TC) than individuals that hatch early in the season (weak TC), and typically express faster growth rates and shorter development times (Johansson et al. 2001; Raczyński et al. 2021).

Adult females were collected at a natural pond in north‐western Poland (53°39′27.6″N, 16°16′26.6″ E) on 10 August 2022 using a sweep net (Sniegula and Johansson 2010). After catching females, they were placed in separate plastic containers with wet filter paper for egg laying. Females were kept at room conditions with a natural photoperiod and a temperature of 22°C until egg laying, which occurred within 3 days. Out of 37 field‐collected females, 18 laid egg clutches that were then used during the experiment. On 12 August 2022, egg clutches were transported by car to the Institute of Nature Conservation PAS (INC PAS) in Krakow, Poland, where the experiment was carried out.

We reared damselflies in two incubators (Pol‐Eko ST700) with programmed temperatures and photoperiods corresponding to those at the sampling site. Specifically, during the egg phase (experimental summer, fall, and winter period), we simulated the gradual decrease of temperature and photoperiod with weekly intervals. During the egg phase, the water temperature was derived from the Flake model (Lake Model Flake 2009), which corresponds to real water temperature in the shallow section of the sampling site (Wos et al. 2023). During the larval phase (the following spring and summer period), and depending on the TC treatment, we simulated early (weak TC) and late (strong TC) season photoperiods with a weekly interval change. For the details of the applied photoperiod regimes, see Figure S1 and Tables S1 and S2. During the larval phase, the air temperature in incubators was set constant at 24°C, which resulted in a water temperature approximately 1.5°C lower. This water temperature corresponds to the temperature in the shallow section of the sampling pond during the larval growth season (especially during the time when larvae are in later instars), as was shown by logger reads installed at such pond section in 2023 (Figure S2). The temperature was kept constant during the larval phase, as previous studies have shown that seasonal changes in photoperiod induce a stronger influence on the studied traits than corresponding changes in temperature (Moghadam et al. 2019; Neptune and Benard 2024). Note that weak TC and strong TC groups experienced similar absolute values of photoperiod throughout the experiment (Figure S1). However, the weak TC group started with a short daylight matching early spring and thereafter experienced an increase in daylight duration (April to June), whereas the strong TC group started with daylight matching long mid‐summer and thereafter experienced a decrease in daylight duration (July to September). This decrease in photoperiod imposes TC in *L. sponsa* (Norling 2018).

### 2.2 Predator Description, Collection, and Housing

The invasive alien spiny‐cheek crayfish, *Faxonius limosus* , is a North American species introduced to Central Europe in the late 19th century (Bonk and Bobrek 2020; Kouba et al. 2014). Now, it is the most prevalent crayfish species in European countries, including Poland and the specific study area (Kouba et al. 2014; World of Crayfish 2024). It inhabits lakes and rivers near the damselfly sampling locations (Śmietana 2016; Maciej Bonk and Szymon Sniegula unpublished data). It is an omnivorous decapod that preys on aquatic invertebrates, including freshwater insects (Twardochleb et al. 2013). Since it has co‐occurred with our damselfly sampling population for more than several decades, we might expect induced predator effects in *L. sponsa* eggs and larvae (Anton et al. 2020), as previously shown in another damselfly species, *I. elegans* (Antoł and Sniegula 2021; Sniegula et al. 2025; Wos et al. 2023).

We collected crayfish from Kryspinów Reservoir in Southern Poland (50°3′0.46″ N, 19°47′20.85″ E) by hand, Using a net, several weeks before the experiment started. The animals were transported to the INC PAS, where they were held in Aquaria. To minimize the accumulation of nitrogenous waste, which could interfere with prey response to predator cues, aquaria were regularly cleaned and water was frequently refilled. Also, crayfish were kept at low densities (three individuals in 40 L of reconstituted deionized water, RDiW) in aquaria maintained at a constant temperature of 20°C (Chen and Kou 1996). The densities of crayfish in aquaria were Based on previous experiments where the crayfish cues were effectively used to induce antipredator responses in damselfly Larvae (Amer et al. 2024; Wos et al. 2023). Crayfish were Fed with fish food pellets twice per week and alive worms once per week. Water from these aquaria was Used to install the predator cue treatment (described below). Crayfish were field‐collected and held under Laboratory Conditions With permission from the regional directorate for environmental protection in Kraków (Ref. OP.672.4.2021.GZ).

### 2.3 Copper Solution Preparation

The chemical compound, copper sulphate pentahydrate (CuSO_4_.5H_2_O) was obtained from CHEMPUR, PL with purity: 99.9%. We prepared a stock solution of 10 g/L by weighing 0.1 g of CuSO_4_.5H_2_O and mixing it with RDiW water to a volume of 10 mL. From this stock solution, we prepared one test concentration: 2 mg/L in addition to a control. We selected this concentration based on our pilot study, which utilized six different concentrations of CuSO_4_.5H_2_O (0, 1, 3, 5, 10, and 15 mg/L). After analyzing the lethal effects of these concentrations, we found that 2 mg/L corresponds to the LC_35_ (lethal concentration that kills 35% of the population) (Figure S3). Additionally, this concentration falls within the environmental range used in agricultural activities (Lusk and Chapman 2020; Tamm et al. 2022). A volume of 15 mL in triplicate of newly prepared copper concentration and control RDiW water was saved in plastic Falcon tubes at 4°C for chemical analysis. The nominal concentration of CuSO_4_.5H_2_O was calculated to provide the equivalent concentration of 0.508 mg/L Cu^+2^, which was measured and confirmed using an ICP‐MS instrument (Chemical lab, Jagiellonian University, Kraków, Poland) to be 0.502 ± 0.018 mg/L (newly prepared) and 0.586 ± 0.087 (after 2 days) under the following liquid sample digestion procedure. A volume of 120 μL concentration trace metals grade HNO_3_ was added to 1.5 mL of each sample. Samples were introduced to a hot block at 95°C for 30 min. A volume of 75 μL of 30% H_2_O_2_ was added gradually to hot samples (25 and 50 μL were added over 5 min) and heating was continued for an additional 60 min, before removal from the hot block. Samples were allowed to cool and centrifuged. A final volume (FV) = 1.5 mL with MQW was brought up. Final matrix = 8% HNO3.

### 2.4 Experimental Treatments

**FIGURE 1:**
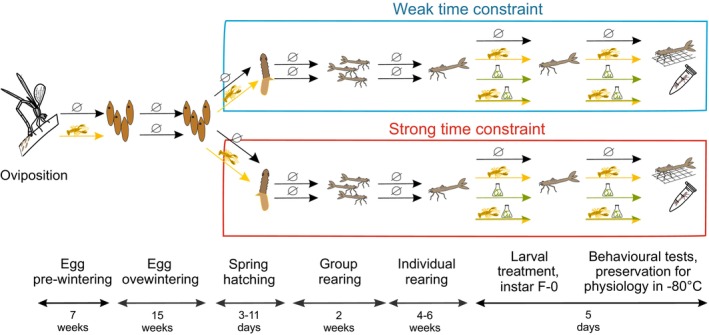
Experimental design. *L. sponsa* eggs were assigned to two egg treatment groups receiving no predator cues (black arrow with crossed circle) and predator cues from invasive alien spiny‐cheek crayfish (yellow arrow with the crayfish symbol). Eggs were overwintered for 15 weeks. Irrespective of the egg predator treatment groups, eggs received no predator cues during wintering period. Time constraint (TC; weak TC and strong TC) treatment was introduced to egg stage directly after overwintering and continued to larval stage (end of experiment). Weak time constraint reflected short (and increasing in time) photoperiod perceived as considerable time to develop as larvae before adult emergence. Strong time constraint reflected long (and decreasing in time) photoperiod, indicating less time for larval development. After hatching, larvae under both TC treatments continued with no predator treatment until reaching the final instar before emergence (F‐0). At the entrance of F‐0, larvae were grouped to four larval‐treatments under each TC treatment and egg predator treatment for 5‐days treatment: No predator—no metal (black arrow), predator—no metal (yellow arrow), no predator—metal (green arrow), and predator—metal (yellow‐green arrow). At day 2 of 5‐days treatment, larvae were assigned to behavioural testing. After behavioural testing (on day 5 following entry into the F‐0), larval growth rate was measured, and larvae were stored at −80°C for subsequent physiological analyses.

In this study, we have two time‐constrained treatments (weak and strong) that run after egg overwintering to the end of the experiment, two egg predator treatments (no predator and predator cue) that run before and after egg overwintering until hatching, and four larval treatments predator cues × copper (no predator—no metal, no predator—metal, predator—no metal, predator—metal) that started when the larvae entered the final instar prior to emergence (F‐0) (Figure 1). We did not expose eggs to predator cues during the wintering period because spiny‐cheek crayfish are largely inactive under cold temperatures (Tricarico 2019) and hence reduce feeding and release minimal cues during winter months.

### 2.5 Egg Treatments

During the egg phase, only the predator treatment was applied to avoid the high mortality rate copper induces in the egg stage (see Amer et al. 2021). Eggs were overwintered (egg diapause) for 15 weeks at 24 h of darkness and 8°C. Overwintering allows eggs to synchronize their life cycle during the following spring and summer (growing and breeding season). Overwintered eggs can resist cold and desiccation, enabling survival until spring. We followed the procedure by Norling (2018) to terminate egg diapause. Temperatures were gradually increased (8°C, 14°C, 18°C, 22°C, and 24°C) for a week and photoperiod was adjusted along with the TC treatments. Hence, TC was introduced after the egg‐wintering period (Figure 1, Figure S1). For each egg clutch, the filter paper containing the eggs was evenly divided into four pieces visually, each allocated to one of the four egg treatments: two egg predator cue treatments (no predator and predator cue), applied before and after egg wintering, crossed with two TC treatments (weak TC and strong TC), applied after egg wintering. Due to the high number of eggs per clutch (often close to or above 100 eggs per clutch), individual eggs were not counted; however, care was taken to ensure approximately equal distribution across treatments. Eggs from every clutch were placed in plastic containers (15 × 11 × 7.5 cm) that corresponded to the four egg treatments. Hereby, clutches were pooled. Each no predator group container was filled with 600 mL of control dechlorinated tap water. For the predator cue group, each container was filled with 400 mL of control dechlorinated tap water and 200 mL of predator cue water. Water refilling of 200 mL of dechlorinated tap water (no predator group) and 200 mL of predator cue water (crayfish‐conditioned water—predator cue group) was scheduled every second day starting on 12 August when egg clutches were placed in the incubator and continued until 30 September when the temperature and photoperiod were switched from 10°C and L‐D 08:53–15:07 to 8°C and L‐D 00:00–24:00, i.e., simulation of winter conditions. After the winter period, refilling resumed on the same schedule and continued until the first larva in each container hatched, after which water refilling was stopped (typically within 3–11 days, see the Results) (Figure 1). This refilling procedure was effective in producing life history patterns in previous experiments on damselfly eggs (Amer et al. 2024; Antoł and Sniegula 2021).

### 2.6 Larval Treatments

At hatching, larvae coming from both egg predator treatment groups (no predator and predator cues) continued as a no predator treatment group up to entering the final instar prior to emergence (F‐0) in both TC treatment groups. This allowed us to check for carryover effects caused by predator cues from egg to F‐0. At hatching, 10 randomly chosen larvae from each treatment were transferred to plastic cups (7.5 cm height × 3.5 cm diameter) and kept in these group cups for another 14 days to increase their survival (De Block and Stoks 2003). To minimize cannibalism, each group cup was fed ad libitum by receiving 1 mL of *Artemia* nauplii solution (N_mean_ = 235, SD = ±28.5 nauplii/ml, *N* = 10) twice a day. It was shown that well‐fed odonate larvae show weak cannibalism (De Block and Stoks 2004; Johansson 1992). Then, larvae were transferred into individual cups and each larva received 1 mL of *Artemia* nauplii. During the whole experiment, larvae were fed twice a day (morning and afternoon) during weekdays and once a day during weekend days, except during the 5‐day larval treatment when they were fed twice a day every day. When larvae entered the pre‐final instar prior to emergence (F‐1), they were given in addition to the *Artemia*, two live chironomid larvae on the first and third days of this instar (the F‐1 instar lasted approx. 10 days under our experimental conditions). When larvae moulted into F‐0s, we started a 5‐day treatment in each of the four combinations of time‐constraint and predator egg treatment groups. We tracked larval instars by detecting exuviae and visually assessing head width and wing pad length. We chose the F‐0 because it represents a key developmental stage. Previous studies have shown that damselfly larvae exhibit considerable mass increase during the first days after entering F‐0 (Jorissen et al. 2023; Palomar et al. 2023). Moreover, during this stage, damselfly larvae show behavioural and physiological plasticity (Johansson et al. 2001; Stoks, De Block, and McPeek 2006; Wos et al. 2025), highlighting the biological relevance of this developmental window. Newly moulted F‐0s were divided into an additional four treatment groups: no predator—no metal, no predator—metal, predator—no metal, and predator—metal. This resulted in 16 treatment combinations (Figure 1).

Newly moulted F‐0s were transferred individually into plastic cups (7.5 cm height × 3.5 cm diameter) filled with 100 mL of RDiW (no predator—no metal group), 100 mL of copper treatment 2 mg/L (predator no—metal yes group), 50 mL of RDiW and 50 mL of predator cue water (predator yes—metal no group), and 50 mL of predator cue water and 50 mL of copper treatment (4 mg/L) to keep the final concentration of copper 2 mg/L (predator yes—metal yes group). The medium in each cup was renewed every second day (day 0, day 2, and day 4) to keep treatment concentration constant. At day 5, each larva was weighed and saved at a −80 freezer for physiological analysis. Since two incubators were used in this experiment, to minimize potential positional effects related to light intensity or temperature, larvae were regularly rotated between the two incubators, and their positions within each incubator (shelf locations) were also changed throughout the experiment. We stopped the experiment directly after treatments at day 5 to focus on immediate stress responses and also from the logistic reason.

### 2.7 Response Variables

Sample sizes for all experimental groups are provided in Table S3. Below, we present only the ranges across groups for each response variable.

#### 2.7.1 Life History Traits

Survival was measured by following the hatched larvae at the individual level as a binary variable after 14 days from hatching in each group cup (range 78–171 larvae), at the entrance into F‐0 (41–116 larvae), and until 5 days after entering F‐0 (i.e., end of the experiment) (8–28 larvae) to track survival throughout the experiment. Development time was measured as the number of days between oviposition and hatching (egg development time in days; range 73–164 eggs) without the overwintering period. We excluded overwintering time from egg development time measurements because it is a physiologically inactive phase during which development is arrested. Growth rate until the entrance into F‐0 was calculated as growth_F‐0_ = mass at the entrance into F‐0/development time between hatching and entrance into F‐0 (41–116 larvae). Larvae were weighed to the nearest 0.1 mg using an electronic balance (Radwag AS.62. R2 Plus). To avoid harm to the newly hatched larvae, we did not measure their initial mass. Yet, given their very small size, their initial mass was considered to be zero. Following Stoks, De Block, and McPeek (2006); Stoks, De Block, Slos, et al. (2006), we used the mass accumulated at the start of the F‐0 stage divided by the duration into the F‐0 as a proxy of cumulative larval growth. This approach follows previous studies on odonates (De Block and Stoks 2003; Johansson and Rowe 1999). Growth rate during the larval treatment was calculated as growth_5‐days_ = (mass after larval‐treatment—mass at the entrance into F‐0)/5 days (8–28 larvae).

#### 2.7.2 Behavioural Traits

We measured four behavioural traits: activity (7–20 larvae), resting time (s) (9–28 larvae), freezing time (9–28 larvae), and feeding rate (9–28 larvae) based on video recordings. Each larva was tested in conditions that matched the original conditions used during the 5‐day experiment, including predator cue and/or copper treatment. For this, on day 2 after the entrance into F‐0, each larva was first placed in an individual, transparent container measuring 12 × 8 × 5 cm. The containers were filled with 200 mL of rearing water containing the same concentration of the experimental stressor (predator cue and/or copper) as in the climate chambers. These containers were kept in a temperature‐controlled laboratory at 24°C, matching the rearing conditions. This setup allowed us to ensure that behavioural responses reflected the effects of experimental stressors. The containers with larvae (one larva per container—up to six containers at a time) were placed under a camera stand for 15 min to allow the larvae to acclimate to the new conditions. Grey cardboards were positioned between individual containers to reduce larval stress and prevent them from observing other individuals. After this period, a camera (Olympus 500D) was activated to record larval movement for 10 min (measuring distance moved, velocity, movement time, and resting time) for further analysis using Ethovision XT 9.1 Software (Noldus Information Technology Inc., Leesburg, VA, USA) (Spink et al. 2001). The recording environment and camera position were standardized across all trials, and the dimensions of the arena were predefined in the software. Subsequently, each larva was gently touched on the thorax using a pencil, and the time the individual remained motionless was measured (freezing time), as in Debecker and Stoks (2019) and Golab et al. (2020). Larvae that have shorter freezing times are considered bolder. Following this, 30 *Artemia salina* nauplii were introduced into each container as food, and another 15‐min session commenced for larval feeding behaviour measuring the number of eaten *A. salina* (# eaten/15 min). After this time elapsed, larvae were removed from the experimental containers and placed back into their original rearing containers in the climatic chamber, while the remaining *A. salina* nauplii in the containers were counted using a hand magnifier.

For the distance moved (cm), the two‐dimensional movement was determined by measuring the movement relative to the centre point of each organism's body. We analysed velocity by measuring the mean speed during the times when larvae were moving. Movement time (seconds) was analysed by calculating the time when larvae were moving. We reduced the number of variables of behavioural traits by averaging the Z‐scores for the distance moved, velocity, and movement time to be included as activity. Resting time was analysed by calculating the time (seconds) when larvae were not moving. Freezing time (seconds) was analysed by calculating the time the individual remained motionless after stimulation with the pencil.

#### 2.7.3 Physiological Traits

We quantified physiological traits on the body supernatants of larvae collected at the end of the five‐day‐long experiment in the F0 stage. To prepare the body supernatants, we homogenized the larvae in PBS buffer (Phosphate Buffered Saline, final mass × 15 μL PBS) and then centrifuged the mixture. To measure the oxidative damage to lipids (amount of malondialdehyde, MDA) (8–27 larvae), we used the thiobarbituric acid assay (TBA assay) based on the modified protocol of Miyamoto et al. (2011). The mixture of 50 μL of supernatant and 50 μL TBA solution (0.4%, in 0.1 M HCl) was incubated at 90°C for 60 min. Then, 165 μL of butanol was added to the mixture, mixed vigorously, and centrifuged at 4000 rpm for 3 min. We filled a 384‐well microtiter plate with 30 μL of the final mixture in triplicate and measured the fluorescence at a wavelength of 530–550 nm. We used the standard curve of 1,1,3,3‐tetramethoxypropane 99%, malondialdehyde bis (dimethyl acetol) 99% to calculate the concentration of MDA.

To estimate the metabolic rate, we quantified the activity of the Electron Transport System (ETS) in the mitochondria; we used the protocol of De Coen and Janssen (2003). We loaded a 384‐well microtiter plate in triplicate with 5 μL of the supernatant and 15 μL buffered substrate solution (0.13 M Tris–HCl, 0.3% Triton X‐100, 1.7 mM NADH, 250 μM NADPH, pH = 8.5). Then, we added 10 μL of iodonitrotetrazolium (INT, 8 mM p‐iodonitrotetrazolium). We measured the increased absorbance of the final product formazan every 30 s for 20 min at 25°C. Formazan concentration was calculated based on the Lambert–Beer law (extinction coefficient 15.9 mM−1 cm^−1^) and then converted into cellular oxygen consumption based on the theoretical stoichiometric relationship: for each 2 μmol of formazan formed, 1 μmol of O_2_ was consumed in the ETS system. The means of the triplicate readings were used for statistical analyses.

Fat content was quantified using a modified protocol of Marsh and Weinstein (1966) by Verheyen et al. (2018) (Verheyen et al. 2018) for damselfly larvae. Small glass tubes were filled with 8 μL of supernatant and 56 μL of concentrated sulphuric acid (100%). Tubes were heated at 150°C for 20 min and then cooled down to add 64 μL of milliQ water. We uploaded a 380‐well microtiter plate with 30 μL of the final mixture per larva in triplicate and measured the absorbance at 490 nm. The means of the three readings were used in the statistical analyses.

To measure the total sugar (glucose + glycogen), we used the described protocol in Stoks, De Block, and McPeek (2006) using the glucose kit from Sigma Aldrich (St. Louis, Missouri, USA). We mixed 5 μL of supernatant, 13 μL milli‐Q water, and 2 μL amyloglucosidase (1 unit/10 μL; Sigma A7420) in a 384‐well microtiter plate. After 30 min of incubation at 37°C, all glycogen is transformed into glucose. We measured the glucose levels by adding 40 μL of glucose assay reagent (Sigma G3293) to each well. We measured the absorbance at 340 nm (Infinite M2000, TECAN) after another incubation period of 20 min at 30°C. We calculated sugar concentration based on a standard curve of known concentrations of glucose and their absorbance. Measurements were done in triplicate and the mean was used for statistical analyses.

We measured protein content in the supernatant of every sample using the method of Bradford (1976). We diluted 1 μL of supernatant in 160 μL milli‐Q water (in quadruplicate) in a flat‐bottom 96‐well plate, then mixed vigorously with 40 μL of Bio‐Rad protein dye reagent (No. 500–0006). Measurements took place in a plate reader at 595 nm after 5 min incubation at 25°C. We calculated protein concentration based on a standard curve of known albumin concentrations (Sigma A2153). Measurements were done in quadruplicate, and the mean was used for statistical analyses.

To obtain the cellular energy allocation (CEA) (8–28 larvae), an estimate of the net energy budget, we integrated various physiological parameters. Following De Coen and Janssen (2003), we calculated CEA as the sum of energy available (Ea; stored in proteins, sugars, and lipids) divided by the energy consumed (Ec; estimated from ETS activity), according to Verheyen and Stoks (2020). We first converted the fat, sugar, and protein contents into their respective energetic contents using their combustion energies: 39,500 mJ.mg^−1^ for lipids, 17,500 mJ.mg^−1^ for sugars, and 24,000 mJ.mg^−1^ for proteins (De Coen and Janssen 2003). The ETS activity was then converted using the oxyenthalpic equivalents for an average mixture of lipids, sugars, and proteins, which equals 484 kJ.mol O_2_ (De Coen and Janssen 2003). Lower CEA values indicate less energy available and/or higher energy expenditure, suggesting reduced energy for growth and reproduction. Damselfly larvae with higher CEA values have been shown to exhibit higher growth rates (Verheyen and Stoks 2020).

### 2.8 Statistical Analyses

For statistical analyses, we used R version 4.0.3 (R Development Core Team 2023). Normality was checked for continuous variables, and log transformation was applied to MDA and CEA. For each trait, we initially fitted a full GLM model including all fixed effects and their interactions. Then, we ran a model selection analysis (MuMIn package; Bartoń 2024) to select the most appropriate model and keep only the relevant variables and interactions. For the model selection analysis, we included in the initial model the following factors: TC (weak TC and strong TC), predator egg Treatment (no predator and predator cues), metal larval‐treatment (no and yes), predator Larval‐treatment (No and Yes), sex, and all possible interactions. Results of the full models prior to AIC selection are provided in Table S4. Selection of the best model was made based on the corrected Akaike's information criteria for small sample size (AICc) and weights (Table S5). Next, we ran univariate statistics using generalized linear model (GLM) for each trait based on the output of the model selection analysis (Table S5). After model fitting, residuals were inspected using Q‐Q plots to confirm normality. All final models met the assumptions of normality and homogeneity of variance. For survival until 14 days, until F‐0, and after larval‐treatment, we used a generalized linear mixed model with a binomial distribution with group Cups as a random factor (function glmmTMB; Brooks et al. 2024). For egg development Time, resting time, freezing time, and feeding rate traits, we used a poisson distribution. For the other variables (growth rate, activity, MDA, and CEA), we used generalized linear model (GLM) with a gaussian distribution. The Anova() function from the *Car* package (Fox and Weisberg 2019) was used to compute *p*‐values, followed by pairwise comparisons using Tukey's HSD contrasts as post hoc tests (Emmean Package, Lenth et al. 2020).

## 3 Results

### 3.1 Life History Traits

In general, our analyses indicated that TC had the strongest effect across the different developmental stages on the life‐history traits, followed by the predator cue treatment applied during the egg stage, while the effect of metal and/or predator cues applied at the larval stage was non‐significant.

**FIGURE 2:**
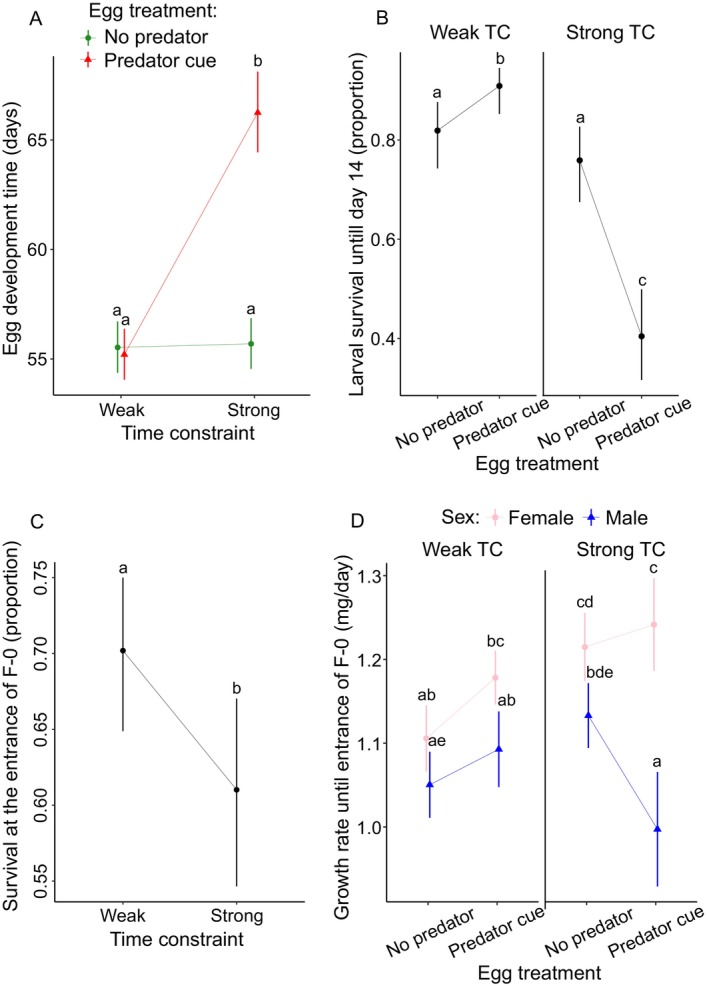
Effect of egg treatment (no predator and predator cue), time constraint (weak and strong), and sex (male, M, and female, F) on egg development time (A), survival until 14 days after hatching (B), survival at F‐0 instar (C), and growth rate until entrance into F‐0 instar (D) in *L. sponsa*. Error bars show 95% CI.

We detected a significant interaction between predator egg treatment × TC, the predator cue experienced during egg stage delayed egg development time, but only in the strongly TC group (Figure 2A, Table 1; sample size range 73–164).

**TABLE 1:** Results of the univariate analyses on the effects of egg treatment (no predator and predator cue), time constraint (weak and strong), sex and their interactions (if significant) on egg development time, larval survival 14 days after hatching and until F‐0 instar, and growth rate until entering the F‐0 instar in *L. sponsa*.

Predictor	df	*X* ^2^	*p*
Egg development time			
Egg treatment Time constraint Egg treatment × time constraint	1 1 1	6.67e^−01^ 1.53e^−05^ 2.50e^02^	0.678 0.414 < 0.001***
Larval survival 14‐days after hatching			
Egg treatment Time constraint Egg treatment × time constraint	1 1 1	27.973 1.379 25.644	< 0.001*** 0.240 < 0.001***
Larval survival until F‐0			
Time constraint	1	5.039	0.024*
Growth rate from hatching until F‐0			
Egg treatment Time constraint Sex Egg treatment × time constraint Time constraint × sex Egg treatment × sex Egg treatment × time constraint × sex	1 1 1 1 1 1 1	2.057 14.545 37.670 9.726 5.511 5.984 3.961	0.152 < 0.001*** < 0.001*** 0.002** 0.019* 0.015* 0.047*

*Note:* The analyses were limited to the relevant predictors and interactions determined by the model selection analysis (AICc). Significant *p*‐values are in bold: *p < 0.05, **p < 0.01, ***p < 0.001.

For larval survival after 14 days, the model selection revealed significant predator egg treatment, TC, and their interactions. Under weak TC, egg treatment showed a positive carry‐over effect on larvae by increasing survival, and under strong TC, egg treatment showed a negative carry‐over effect on larvae by decreasing survival. TC alone did not show an effect on larval survival (Figure 2B, Table 1 and Table S6; sample size range 78–171).

For larval survival at the entrance into F‐0, the best‐fitted model returned only one significant variable, TC, and did not include the predator treatment. Strong TC led to a decreased survival rate when compared to weak TC at the entrance into F‐0 (Figure 2B, Table 1, sample size range 41–116).

For growth rate, the best fitted model indicated that several interactions involving TC, sex, and predator cue exposure were significant (Table 1). Due to these interactions, the main effects of TC and sex should be interpreted with caution, as their effects on growth rate varied depending on the level of other factors. Specifically, the interaction between predator egg treatment and TC showed that exposure to predator cues during the egg stage reduced the growth rate, but only under strong TC (Figure 2C, Table 1, sample size range 41–116). The interaction between TC and sex revealed that males exhibited a decreased growth rate under predator egg treatment, but only when reared under strong TC (Figure 2C, Table 1). The interaction between the predator egg treatment and sex showed that predator cues increased the growth rate, but only in females (Figure 2C, Table 1). A three‐way interaction between TC, predator egg treatment, and sex further demonstrated that predator cues in the larval stage decreased growth rate, but only in males reared under strong TC (Figure 2C, Table 1).

**FIGURE 3:**
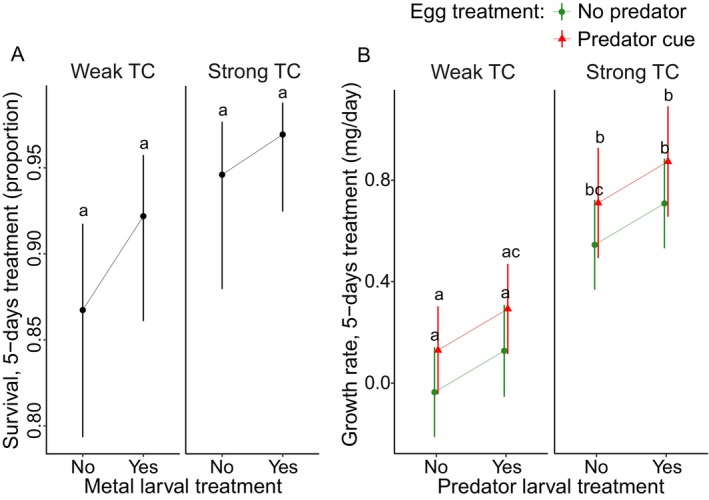
Effect of egg treatment (no predator and predator cue), time constraint (weak and strong), and predator larval treatment (no/yes) on larval survival during 5‐day treatment (A) and growth rate (B) in *L. sponsa*. Error bars show 95% CI.

During larval treatment, individuals from the strong TC group showed higher survival than the weak TC group (Figure 3A, Table 2; sample size range 8–28). The metal treatment had no significant effects. During the larval treatment, strong TC led to faster growth than weak TC (Figure 3B, Table 2; sample size range 8–28). Individuals showed a trend for increased growth rate in response to the larval predator cues (*p* = 0.079, Table S6).

**TABLE 2:** Results of the univariate analyses on the effects of egg treatment (no predator and predator cue), time constraint (weak and strong), predator larval treatment, and their interactions, if significant, on larval survival and growth rate after 5 days of treatment in *L. sponsa*.

Predictor	df	*X* ^2^	*p*
Larval survival after 5‐days treatment			
Time constraint Metal larval treatment	1 1	5.043 2.238	0.024* 0.134
Growth rate during 5‐days treatment			
Egg treatment Time constraint Predator larval treatment	1 1 1	2.924 35.942 3.096	0.089 < 0.001*** 0.079

*Note:* The analyses were limited to the relevant predictors and interactions determined by the model selection analysis (AICc). Significant *p*‐values are in bold: *p < 0.05, ***p < 0.001.

A trend of the predator egg treatment was detected (*p* = 0.089, Table S6) with a faster growth rate in individuals that experienced predator cues during the egg stage.

### 3.2 Behavioural Traits

Next, we tested for the effects of egg treatment, TC, metal larval treatment, predator larval treatment, and their interactions on the response of each behaviour trait separately.

**FIGURE 4:**
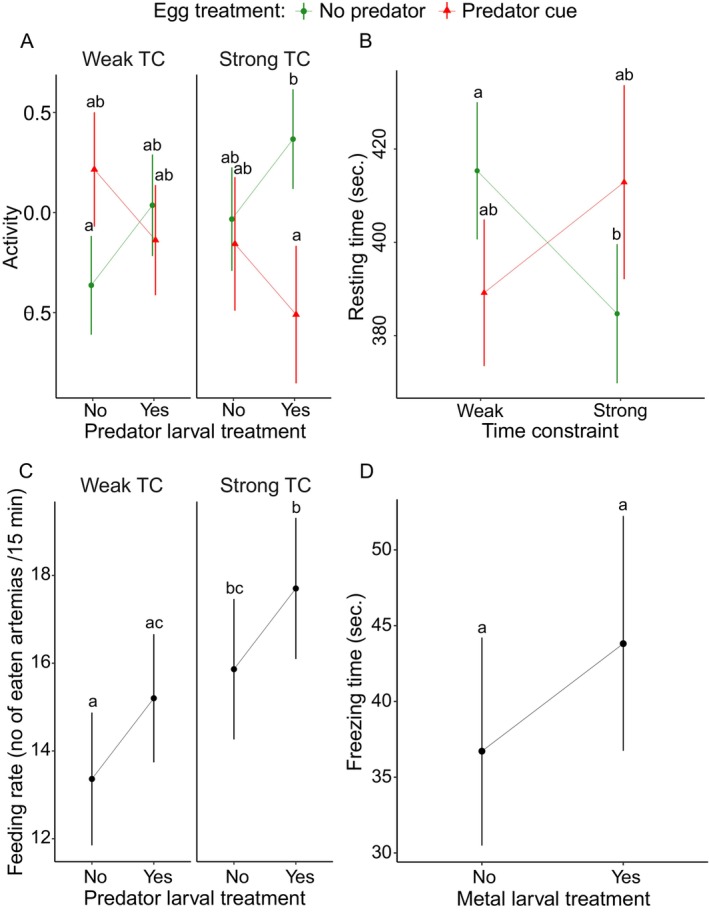
Effect of egg treatment (no predator, predator cue), time constraint (weak and strong), predator larval treatment (yes/no), and metal larval treatment (yes/no) on larval behavioural traits; activity (A), resting time (B), feeding rate (C), and freezing time (D) in *L. sponsa*. For freezing time (D), the effect of the metal larval treatment is shown, as this factor was retained in the second best‐fitting model based on AICc. Error bars show 95% CI.

Exposure to predator cues during larval treatment showed decreased larval activity but only under strong TC (Figure 4A, Table 3; sample size range 7–20). Exposure to predator cues during the egg stage increased activity but only under weak TC in the absence of exposure to predator cues during the larval treatment (Figure 4A, Table 3). Egg predator cues increased resting time under strong TC but reduced it in weakly TC individuals (Figure 4B, Table 3; sample size range 18–28). Strong TC increased feeding rate. Predator larval treatment showed increased feeding rate (Figure 4C, Table 3; sample size range 9–28). Freezing time was affected by none of the variables (Figure 4D, Table S6; sample size range 9–28).

**TABLE 3:** Results of the univariate analyses on the effects of egg treatment (no predator and predator cue), time constraint (weak and strong), and predator larval treatment on the behavioural traits: Activity, resting time (seconds), and feeding rate (number of eaten *Artemia*/15 min) in *L. sponsa*.

Predictor	df	X^2^	*p*
Activity			
Egg treatment Time constraint Predator larval treatment Egg treatment × predator larval treatment Egg treatment × time constraint	1 1 1 1 1	9.133 5.005 7.324 10.628 8.832	0.002** 0.026* 0.007** 0.001** 0.003**
Resting time			
Egg treatment Time constraint Egg treatment × time constraint	1 1 1	5.732 8.312 10.255	0.017* 0.004** 0.001**
Feeding rate			
Time constraint Predator larval treatment	1 1	7.578 4.148	0.006** 0.042*

*Note:* The analyses were limited to the relevant predictors and interactions determined by the model selection analysis (AICc). Significant *p*‐values are in bold: *p < 0.05, **p < 0.01, ***p < 0.001.

### 3.3 Physiological Traits

We tested for the effects of predator egg treatment, TC, metal larval treatment, predator larval treatment, and their interactions on the response of each physiological trait separately.

**FIGURE 5:**
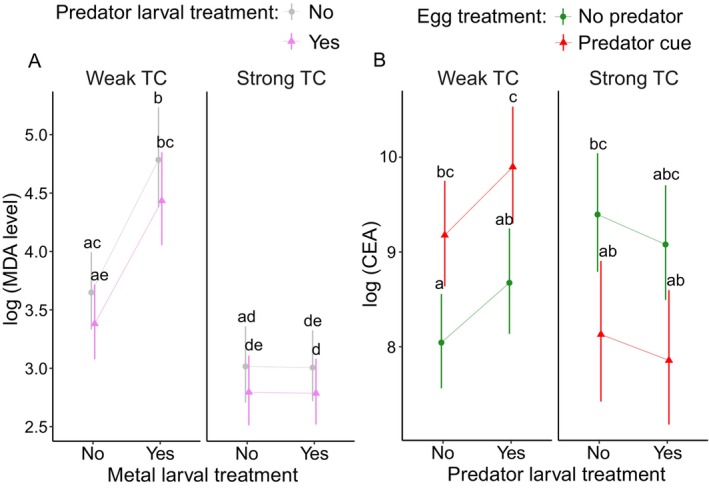
Effects of egg treatment (no predator and predator cue), time constraint (weak TC and strong TC), metal larval treatment (no/yes), and predator larval treatment (no/yes) on oxidative damage (MDA) (A) and cellular energy allocation (CEA) (B) in *L. sponsa*. Error bars show 95% CI.

We found significant effects of TC and metal larval treatment with an increased MDA under weak TC. The interaction metal larval treatment × TC was significant with an increase in MDA when exposed to metal, but only under weak TC (Figure 5A, Table 4 and Table S5; sample size range 8–27). Predator larval treatment showed a trend for decreased MDA levels (Figure 5A, Table 4 and Table S6).

**TABLE 4:** Effects of egg treatment (no predator and predator cue), time constraint (weak and strong), metal larval treatment, predator larval treatment, and sex on the physiological traits: Oxidative damage (MDA) and cellular energy allocation (CEA) in *L. sponsa*.

Predictor	df	*X* ^2^	*p*
MDA (oxidative damage)			
Time constraint Metal larval treatment Predator larval treatment Metal larval treatment × time constraint	1 1 1 1	57.87 22.109 2.995 9.448	< 0.001*** < 0.001*** 0.084 0.002**
CEA (cellular energy allocation)			
Egg treatment Time constraint Predator larval treatment Predator larval treatment × time constraint Egg treatment × time constraint	1 1 1 1 1	13.066 11.324 4.281 3.725 20.0.994	< 0.001*** < 0.001*** 0.039* 0.054 < 0.001***

*Note:* The analyses were limited to the relevant predictors and interactions determined by the model selection analysis (AICc). Significant *p*‐values are in bold: *p < 0.05, **p < 0.01, ***p < 0.001.

Exposure to predator cues during the egg treatment increased CEA in a weak TC group and decreased CEA in a strong TC group (Figure 5B, Table 4 and Table S6; sample size range 8–28). Predator larval treatment showed a trend for increased CEA, but only in the weak TC group (Figure 5B, Table 4).

## 4 Discussion

While we detected several two‐way stressor interactions, including stressor interactions across different life stages, in contrast with the emerging pattern (Diamant et al. 2023), we did not detect that any of these two‐way interactions were modulated by the third stressor. Exposure to predator cues in the egg stage, not only affected the egg stage but also caused carry‐over effects in the larval stage by shaping behavioural (larval activity) and physiological (cellular energy allocation, CEA) traits. As expected, in response to the TC treatment, larvae increased growth rate. The exposure to the used metal concentration had limited effect and only increased oxidative damage to lipids (MDA), yet only under the weak time constraint. Moreover, the TC treatment modified the effects of exposure to predator cues in the egg stage on larval behaviour (activity, resting time and feeding rate) and physiology (CEA). These findings underline the importance of focusing on organisms' responses at different developmental stages and incorporating seasonality into multiple stressor studies to better understand how multiple stressors interact in natural ecosystems.

### 4.1 Effects of Predator Cues During the Egg Stage and TC on Life History Traits Until the F‐0 Stage

Eggs exposed to predator cues delayed their development under strong TC rather than compensating for the late season date by shortening egg development. Delayed egg hatching under predator cues may stem from physiological costs associated with predator‐induced stress, such as increased energy demands for repair or defense mechanisms (Hawlena and Schmitz 2010). Similar results were shown in the damselfly *Enallagma cyathigerum* eggs after being exposed to fish predator cues (Sniegula et al. 2019), and in the damselfly *I. elegans* eggs after being exposed to cues from native and invasive alien crayfish species (Amer et al. 2024; Sniegula et al. 2025). On the other hand, prolonged egg development time under predation risk might function as a defensive strategy, reducing the chance of hatching into a high‐risk environment (Benard 2004), as demonstrated in the frog *Agalychnis callidryas* (Warkentin 2011).

The analysis of traits during the F‐0 stage provided evidence for carry‐over effects of exposure to predator cues during the egg stage, particularly for growth rate. Weakly TC individuals under predation stress during the egg stage showed faster growth rates compared to their control counterparts. This increased growth might represent an adaptive response to predator cues. Fast‐growing larvae can reach a less vulnerable larval size and developmental stage (terrestrial adult) more quickly. Similar predator‐induced growth responses have been observed in other systems, where temperate butterfly and damselfly larvae under weak or no time stress prioritized growth rate acceleration over acute predation risk (Gotthard 2000; Stoks et al. 2012). Interestingly, male larvae originating from predator‐exposed eggs expressed reduced growth rates under strong TC, suggesting sex‐specific responses due to hormonal regulation or behavior, where males might be more sensitive to environmental cues, especially under stress. Such variation in energy allocation under predator stress was shown in other damselfly species from the family Coenagrionidae (e.g., Stoks, De Block, and McPeek 2005). No such response was found in females. These sex‐specific differences may be due to variations in energy investment for reproduction. Given that reproduction is energetically costlier to females than males (Scharf et al. 2013), females may express a weaker antipredator response and be less plastic than males to preserve the energy needed for reproduction. A similar pattern was documented in the snail *Nucella lapillus* , where a growth rate suppression in response to predation risk occurred in males only (Donelan and Trussell 2020).

Larval survival until day 14 did not decrease when exposed to strong TC alone, indicating the absence of a lethal TC effect during early larval development. However, larvae under strong TC suffered lower survival when exposed to predator cues during the egg stage, whereas larvae experiencing weak TC showed the opposite pattern. These opposing carry‐over effects suggest alternative strategies in dealing with two co‐occurring stressors. Larvae under weak TC may have invested more in antipredator defenses, such as behavioral changes or physiological shifts, potentially at the cost of growth and energy reserves, whereas larvae experiencing strong TC may have prioritized rapid growth and development to complete their life cycle before environmental conditions deteriorate. A similar response was earlier shown in a confamiliar damselfly *C. viridis* (Stoks, De Block, Slos, et al. 2006). These results suggest that TC influences how larvae respond to early predator cues, which aligns with general predator–prey and time allocation theories (Dmitriew 2011). When reaching the F‐0, survival rate was influenced by TC, with lower survival observed under strong TC regardless of predator egg exposure. This may be due to the energetic demands of rapid growth under such conditions (McPeek 2004), as earlier indicated in other ectotherms (Gotthard 2000; Wang, Atlihan, et al. 2022), including damselflies (Dańko et al. 2017; Stoks, De Block, Van de Meutter, and Johansson 2005). Interestingly, we did not find carry‐over effects of predator egg exposure on survival until F‐0, suggesting that predator‐induced stress during the egg stage may have more pronounced carry‐over effects on physiology and behavior rather than on larval survival in older larvae (discussed below). This agrees with previous findings that predator cues often trigger non‐lethal shifts in prey traits (Palacios and McCormick 2021; Urban 2007). Together, these results indicate that carry‐over effects from egg to larval stages are trait‐ and sex‐dependent, with growth rate being more affected by early predator exposure than survival until F‐0.

### 4.2 Effects of Stressors During the Final Larval (F‐0) Instar

A weak larval response to exposure to predator cues in F‐0 can reflect developmental stage‐specific sensitivity. Although the spiny‐cheek crayfish is common in the study area (Kouba et al. 2014), and previous studies on the damselfly *I. elegans* show relatively weak gene expression responses to its cues (Wos et al. 2024), this weak response may be adaptive. Early life stages, like eggs, which are immobile and highly vulnerable, often exhibit stronger antipredator responses than more mobile larval stages that possess behavioural and physiological defences (Ferrari et al. 2010), as previously shown in *I. elegans* , where eggs were more responsive to crayfish predation cues than larvae (Amer et al. 2024; Sniegula et al. 2025). A study on tree frogs also showed that eggs exposed to snake attacks responded more strongly by hatching rapidly to escape into the water, whereas larvae exposed to aquatic predators showed less pronounced responses. This adaptive response aligns with the lower vulnerability of tadpoles to aquatic predators (Warkentin 1995) and, more broadly, illustrates how vulnerability shapes the magnitude of antipredator responses across life stages.

Life‐history and behavioural traits were mainly shaped by TC and predator cues during the egg (carry‐over effects) and larval stages. Increased growth rate under strong TC was accompanied by increased larval activity and feeding rate, and decreased resting time, but only in larvae not exposed to predator cues during the egg stage. These behavioural adjustments are likely adaptive because elevated growth rates require a higher food acquisition. However, the presence of predator cues in the egg stage may offset behavioural compensation (Westwick and Rittschof 2021), indicating that larvae prioritize predator avoidance over maximizing growth‐related behaviours in risky environments. Similar results were shown in other studies. For example, *Enallagma* damselfly larvae previously exposed to predator cues showed a significant reduction in activity even when they were also under strong time stress (McPeek and Peckarsky 1998). Contrary to our F‐0 results, sex‐specific differences were more pronounced earlier in development, suggesting that as larvae approach metamorphosis, both sexes may converge in growth patterns due to shared physiological demand associated with the transition to the adult stage (Minelli and Fusco 2010; Stoks and Córdoba‐Aguilar 2012).

Larvae treated with predator cues during the egg stage showed an increased net energy budget, as measured by cellular energy allocation (CEA), but only in the weakly time‐constrained group, leading to a positive carry‐over effect. This suggests that early exposure to predator cues under less stressful abiotic conditions may increase larval physiological functioning, potentially by better allocation of energy for dealing with predator‐related stress. Our results contrast with a study showing that larval exposure to predator cues caused a direct reduction in CEA in the damselfly *E. cyathigerum* (Van Dievel et al. 2019). Predator stress is often associated with increased metabolic rate and mobilization of energy reserves in prey (Clinchy et al. 2013; Hawlena and Schmitz 2010). This may indicate higher investment into repair mechanisms and building new tissues.

Interestingly, strongly time‐constrained larvae showed higher survival than those from the weak TC group. The higher survival during larval treatment under strong TC might be influenced by earlier selective mortality in this group. Since survival until F‐0 was lower (high mortality) under strong TC, it is likely that individuals entering F‐0 were more stress tolerant or physiologically robust, which may explain the observed increase in survival during the subsequent larval treatment (van de Pol et al. 2006). Moreover, strongly time‐constrained larvae showed decreased CEA after exposure to predator cues during the egg stage, indicating a negative carry‐over effect. This suggests that the combined pressures of strong TC and predation risk drive larvae to reallocate energy toward growth and development rather than maintaining a high energy budget. These findings support our observation of increased growth rates under time stress. Similarly, previous studies on larvae of the damselfly *L. sponsa* under strong TC and predation risk demonstrated that individuals allocated energy to accelerate growth and development (Johansson et al. 2001). Our results add to the knowledge that such energy reallocation in the larval stage can be caused by current and previous stressors, such as TC and predation risk during the egg stage, and that these stressors can interact to influence energy allocation strategies in aquatic ectotherms.

Contrary to our expectations, metal exposure during the F‐0 stage had no effect on the life‐history traits and behavioural responses we measured. One possible explanation for the absence of response might be that larvae have evolved compensatory mechanisms to overcome the negative effects of metals, like reallocating energy resources and/or activating detoxification pathways to minimize the toxicity of metal (Nordberg et al. 2014). Such a compensatory mechanism was shown in moth *Spodoptera litura* larvae where glutathione S transferase eliminated the oxidative stress induced by several insecticides and heavy metals (Xu et al. 2015). The absence of any behavioural responses to copper contrasted with previous studies. Copper, as other trace metals, is indeed known to decrease a prey's ability to detect predators by inhibiting the expression of olfactory genes; hence, the effect should appear on behavioural responses (Amer et al. 2022; Hayden et al. 2015). For example, copper was shown to decrease a prey's ability to detect predator cues (McIntyre et al. 2012; Van Ginneken et al. 2018). In line with this, exposure to copper increased the vulnerability to predation of caddisflies *Hydropsyche morose* (Clements et al. 1989), the polychaete *Nereis diversicolor*, and the bivalve *Scrobicularia plana* (Bonnard et al. 2009). A possible reason for this discrepancy is the relatively short exposure time in our experiment (only 5 days), which may have been insufficient to elicit detectable responses, especially if such effects accumulate more gradually.

Another key finding was that metal exposure had a strong effect at the physiological level that crucially depended on the time constraints. Indeed, copper increased oxidative damage to lipids (measured as MDA), but only in individuals exposed to weak TC. The finding that copper did increase oxidative damage to lipids in larvae not exposed to time constraints may have important fitness implications, including a shortened adult life span (Janssens and Stoks 2018). Under stronger TC, we observed consistently low levels of MDA, also in the presence of copper. Copper is known to induce oxidative stress (Paithankar et al. 2021), resulting in increased levels of Reactive Oxygen Species (ROS) and lipid peroxidation, for example shown in *Drosophila melanogaster* (Saudi et al. 2022). In the current study, higher levels of MDA in the weak TC group when exposed to copper did not seem to be linked with life‐history adjustments as growth rate was generally unaffected by copper. However, we observed a uniform decrease in MDA in strong TC larvae, which might result from increased physiological protection against oxidative stress and lipid peroxidation. Time‐constrained individuals may optimize their energy allocation by changing investment into non‐essential processes, such as immune responses, towards growth and development. This optimization can lead to a more efficient metabolic process, increasing antioxidant protection and consequently lowering the oxidative damage (Dmitriew 2011; Zera and Harshman 2001). In turn, this can lead to the expression of fast compensatory growth with decreased mortality, as seems to have occurred in the current study. Another relevant study indicated that time‐stressed damselfly *Chalcolestes viridis* larvae exposed to DNP (the mitochondrial uncoupler 2,4‐dinitrophenol) that caused a reduced production of reactive oxygen species (ROS), developed faster and did not show an increase in oxidative damage to lipids (MDA) (Janssens and Stoks 2018). Also, time‐stressed *D. melanogaster* showed high resistance to oxidative damage from methyl viologen, a standard oxidative stress test (Harshman and Haberer 2000). Similarly, more time‐stressed *I. elegans* larvae showed lower oxidative damage, which was associated with higher antioxidant defence, suggesting that the accelerated life history and the associated increased production of ROS caused an overcompensatory antioxidant response (Tüzün et al. 2020). This pattern is consistent with hormesis—a process in which low‐level stress stimulates protective physiological mechanisms (Costantini et al. 2010). These results reflect the ability of insects to manage oxidative stress effectively under TC conditions by increasing antioxidant levels.

## 5 Conclusion and Implications

Our results highlight that stressors such as time constraints (TC), predation risk, and copper exposure interact in complex and stage‐dependent ways, shaping damselfly life history, behaviour, and physiology. These interactions are not uniformly negative or additive; rather, they produce both immediate and carry‐over effects that differ across developmental stages and trait types. However, despite an experimental design explicitly incorporating three stressors to test for three‐way interactions, we found no statistical support for such higher‐order interactions. This absence suggests that while pairwise interactions can be strong, their modulation by a third stressor may be context‐dependent or more subtle than detectable within the scope of our experiment.

Our study adds to the emerging concern that effects of pollutants need to be considered in an ecologically relevant context in the presence of natural stressors (Burton & Rohr 2025). Indeed, copper exposure, for instance, had limited direct effects on survival or behaviour but significantly altered physiological responses depending on TC, suggesting that traditional ecotoxicological assays may underestimate toxicity under ecologically realistic conditions. Our results further underscored the importance of delayed effects across life stages. Indeed, predator cues experienced during the immobile egg stage modified behaviour and energy allocation in the mobile larval stage, demonstrating long‐term consequences of early‐life stress. Such findings are especially relevant to organisms with complex life cycles, where stage‐specific sensitivity and carry‐over effects may shape not only immediate fitness components but also long‐term developmental trajectories and life history strategies.

Overall, our results provide important evolutionary insights by showing that developmental plasticity in response to multiple environmental stressors may allow organisms to buffer stress effects, but potentially at a cost to other functions. The trait‐ and stage‐specific nature of these responses suggests the potential for differential selection pressures across life stages, indicating how stressor exposure can influence the evolution of plasticity, stress tolerance, and developmental timing. Our study underscores the importance of integrative, cross‐stage approaches in evolutionary ecology and applied ecotoxicology.

## Conflicts of Interest

The authors declare no conflicts of interest.

## Supporting information


**Appendix S1:** eva70169‐sup‐0001‐AppendixS1.docx.

## Data Availability

The data that support the findings of this study are openly available in Dryad at http://doi.org/10.5061/dryad.9ghx3ffx7.
