# Supplementary material for: Time Constraints Modulate the Effects of Predator Cues and a Metal Across Life Stages in a Damselfly
Source: Evol Appl. 2025 Oct 31;18(11):e70169. doi: 10.1111/eva.70169 (PMC12577438; doi:10.1111/eva.70169)
Supplement: Supplementary file 1 — Appendix S1: eva70169‐sup‐0001‐AppendixS1.docx. [file EVA-18-e70169-s001.docx]

**Time constraints modulate the effects of predator cues and a metal across life stages in a damselfly**

**Table S1** The values of week experimental, date in nature, date of experimental, number of light hours, number of dark hours, and temperatures in a **weak time constraint** eggs indicated by relatively short photoperiod at the start of post-wintering treatment. In the experiment, eggs were overwintered, but the overwintering was shortened compared to nature. Therefore, during post-winter treatment, the dates for experimental values did not follow the current values in nature at a particular time point (Figure S1).

| **Week, experimental** | **Date, nature** | **Date, experimental** | **Light hours** | **Dark hours** | **Temperature °C** |
| --- | --- | --- | --- | --- | --- |
| 0 | 12 August, 2022 | 12 August, 2022 | 16:30 | 07:30 | 20.8 |
| 1 | 19 August, 2022 | 19 August, 2022 | 15:59 | 08:01 | 20.6 |
| 2 | 26 August, 2022 | 26 August, 2022 | 15:28 | 08:32 | 19.7 |
| 3 | 02 September, 2022 | 16 September, 2022 | 13:56 | 10:04 | 15.8 |
| 4 | 09 September, 2022 | 14 October, 2022 | 11:57 | 12.03 | 10.6 |
| 5 | 16 September, 2022 | 18 November, 2022 | 09:50 | 14:10 | 10 |
| 6 | 23 September, 2022 | 23 December, 2022 | 08:53 | 15:07 | 10 |
| 7 | 30 September, 2022 | 06 January, 2023 | 00:00 | 24:00 | 8 |
|  | **Overwintering period** | | | |  |
| 22 | 13 January, 2023 | 31 March, 2023 | 14:03 | 9:57 | 8 |
| 22 |  |  | 14:03 | 9:57 | 14 |
| 22 |  |  | 14:03 | 9:57 | 18 |
| 22 |  |  | 14:03 | 9:57 | 22 |
| 23 | 20 January, 2023 | 07 April, 2023 | 14:35 | 9:25 | 24 |
| 24 | 27 January, 2023 | 14 April, 2023 | 15:05 | 8:55 | 24 |
| 25 | 03 February, 2023 | 21 April, 2023 | 15:38 | 8:22 | 24 |
| 26 | 10 February, 2023 | 28 April, 2023 | 16:08 | 7:52 | 24 |
| 27 | 17 February, 2023 | 05 May, 2023 | 16:39 | 7:21 | 24 |
| 28 | 24 February, 2023 | 12 May, 2023 | 17:09 | 6:51 | 24 |
| 29 | 03 March, 2023 | 19 May, 2023 | 17:36 | 6:24 | 24 |
| 30 | 10 March, 2023 | 26 May, 2023 | 18:01 | 5:59 | 24 |
| 31 | 17 March, 2023 | 02 June, 2023 | 18:23 | 5:37 | 24 |
| 32 | 24 March, 2023 | 09 June, 2023 | 18:39 | 5:21 | 24 |
| 33 | 31 March, 2023 | 16 June, 2023 | 18:48 | 5:12 | 24 |

**Table S2** The values of week experimental, date in nature, date of experimental, number of light hours, number of dark hours, and temperatures in a **strong time constraint** eggs indicated by relatively long photoperiod at the start of post-wintering treatment. In the experiment, the overwintering was shortened compared to nature. Therefore, during post-winter treatment, the dates for experimental values did not follow the current values in nature at a particular time point (Figure S1).

| **Week, experimental** | **Date, nature** | **Date, experimental** | **Light hours** | **Dark hours** | **Temperature °C,** |
| --- | --- | --- | --- | --- | --- |
| 0 | 12 August, 2022 | 12 August, 2022 | 16:30 | 07:30 | 20.8 |
| 1 | 19 August, 2022 | 19 August, 2022 | 15:59 | 08:01 | 20.6 |
| 2 | 26 August, 2022 | 26 August, 2022 | 15:28 | 08:32 | 19.7 |
| 3 | 02 September, 2022 | 02 September, 2022 | 13:56 | 10:04 | 15.8 |
| 4 | 09 September, 2022 | 09 September, 2022 | 11:57 | 12.03 | 10.6 |
| 5 | 16 September, 2022 | 16 September, 2022 | 09:50 | 14:10 | 10 |
| 6 | 23 September, 2022 | 23 September, 2022 | 08:53 | 15:07 | 10 |
| 7 | 30 September, 2022 | 30 September, 2022 | 00:00 | 24:00 | 8 |
|  | **Overwintering period** | | | |  |
| 22 | 13 January, 2023 | 23 June, 2023 | 18:51 | 5:09 | 8 |
| 22 | 15 January, 2023 |  | 18:51 | 5:09 | 14 |
| 22 | 17 January, 2023 |  | 18:51 | 5:09 | 18 |
| 22 | 19 January, 2023 |  | 18:51 | 5:09 | 22 |
| 23 | 20 January, 2023 | 30 June, 2023 | 18:47 | 5:13 | 24 |
| 24 | 27 January, 2023 | 07 July, 2023 | 18:36 | 5:24 | 24 |
| 25 | 03 February, 2023 | 14 July, 2023 | 18:19 | 5:41 | 24 |
| 26 | 10 February, 2023 | 21 July, 2023 | 17:57 | 6:03 | 24 |
| 27 | 17 February, 2023 | 28 July, 2023 | 17:31 | 6:29 | 24 |
| 28 | 24 February, 2023 | 04 August, 2023 | 17:04 | 6:56 | 24 |
| 29 | 03 March, 2023 | 11 August, 2023 | 16:34 | 7:26 | 24 |
| 30 | 10 March, 2023 | 18 August, 2023 | 16:04 | 7:56 | 24 |
| 31 | 17 March, 2023 | 25 August, 2023 | 15:32 | 8:28 | 24 |
| 32 | 24 March, 2023 | 01September, 2023 | 15:01 | 8:59 | 24 |
| 33 | 31 March, 2023 | 08 September, 2023 | 14:30 | 9:30 | 24 |
| 34 | 07 April, 2023 | 15 September, 2023 | 13:59 | 10:01 | 24 |
| 35 | 14 April, 2023 | 22 September, 2023 | 13:30 | 10:30 | 24 |
| 36 | 21 April, 2023 | 29 September, 2023 | 12:59 | 11:01 | 24 |

**Table S3** Sample sizes across the experimental groups. The last larval instar before adult emergence is abbreviated as F-0.

| **Predictor** | **Weak time constraint** | | **Strong time constraint** | |
| --- | --- | --- | --- | --- |
|  | Egg No predator | Egg Predator cue | Egg No predator | Egg Predator cue |
| **Life history traits** | | | | |
| **Survival 14 days after hatching** | 153/190 | 171/190 | 142/190 | 78/190 |
| **Survival until F-0 instar** | 103/155 | 116/157 | 103/160 | 41/76 |
| **Survival after larval treatment**  No predator (metal no, predator no)  Metal (metal yes, predator no)  Predator cue (metal no, predator yes)  Metal + predator cue (metal yes, predator yes) | 25/28  21/23  24/26  24/26 | 25/30  22/27  28/30  27/29 | 22/23  25/25  27/29  26/26 | 10/10  8/10  9/10  11/11 |
| **Development time of egg stage** | 155 | 157 | 164 | 73 |
| **Growth rate until F-0** | 102 (51F:51M) | 116 (77F:39M) | 103 (50F:53M) | 41 (24F:17M) |
| **Growth rate during larval treatment**  No predator (metal no, predator no)  Metal (metal yes, predator no)  Predator cue (metal no, predator yes)  Metal + predator cue (metal yes, predator yes) | 25(10F:5M)  21(11F:10M)  24(12F:12M)  24(13F:11M) | 25(15F:10M)  21(17F:4M)  28(17F:11M)  27(19F:8M) | 22(10F:12M)  24(7F:17M)  28(12F:16M)  26(20F:6M) | 10(6F:4M)  8(5F:3M)  8(5F:3M)  10(5F:5M) |
| **Behavioural traits** | | | | |
| **Activity**  No predator (metal no, predator no)  Metal (metal yes, predator no)  Predator cue (metal no, predator yes)  Metal + predator cue (metal yes, predator yes) | 20  17  17  16 | 11  19  14  18 | 14  18  19  16 | 10  9  7  7 |
| **Resting time**  No predator (metal no, predator no)  Metal (metal yes, predator no)  Predator cue (metal no, predator yes)  Metal + predator cue (metal yes, predator yes) | 23  25  22  26 | 18  22  20  28 | 22  28  27  25 | 14  9  9  13 |
| **Freezing time**  No predator (metal no, predator no)  Metal (metal yes, predator no)  Predator cue (metal no, predator yes)  Metal + predator cue (metal yes, predator yes) | 23  25  22  26 | 18  22  20  28 | 22  28  27  25 | 14  9  9  13 |
| **Feeding rate**  No predator (metal no, predator no)  Metal (metal yes, predator no)  Predator cue (metal no, predator yes)  Metal + predator cue (metal yes, predator yes) | 23  25  22  26 | 18  22  20  28 | 22  28  27  25 | 14  9  9  13 |
| **Physiological traits** | | | | |
| **Oxidative damage (MDA)**  No predator (metal no, predator no)  Metal (metal yes, predator no)  Predator cue (metal no, predator yes)  Metal + predator cue (metal yes, predator yes) | 25  21  20  21 | 25  25  21  25 | 20  27  24  25 | 11  9  8  11 |
| **Cellular energy allocation (CEA)**  No predator (metal no, predator no)  Metal (metal yes, predator no)  Predator cue (metal no, predator yes)  Metal + predator cue (metal yes, predator yes) | 25  24  21  24 | 24  28  22  26 | 19  28  25  25 | 11  8  8  12 |

**Table S4.** Results of generalized linear models included all variables and interactions on life-history traits up to the final instar before emergence (F-0) (larval survival 14 days after hatching and until F-0, egg development time, and growth rate until F-0), with predictors egg treatment, time constraint, and sex; and responses during the larval treatment (survival after larval treatment, growth rate during larval treatment, activity, resting time, freezing time, feeding rate, MDA and CEA), using predictors egg treatment, time constraint, metal larval treatment, predator larval treatment, and sex in *L. sponsa.* Significant p-values are in bold: p < 0.05.

| **Life history traits** | | | |
| --- | --- | --- | --- |
| **Predictor** | **df** | **X^2^** | **p-value** |
| **Survival 14-days after hatching** | | | |
| Egg treatment | 1 | 27.973 | **<0.001** |
| Time constraint | 1 | 1.379 | 0.240 |
| Egg treatment x time constraint | 1 | 25.643 | **<0.001** |
| **Survival until F-0** | | | |
| Egg treatment | 1 | 0.382 | 0.536 |
| Time constraint | 1 | 0.922 | 0.337 |
| Egg treatment x time constraint | 1 | 2.780 | 0.095 |
| **Survival after larval treatment** | | | |
| Egg treatment | 1 | 1.105 | 0.293 |
| Time constraint | 1 | 0.210 | 0.646 |
| Metal larval treatment | 1 | 0.018 | 0.892 |
| Predator larval treatment | 1 | 1.104 | 0.292 |
| Sex | 1 | 1.634 | 0.201 |
| Time constraint x Egg treatment | 1 | 0.708 | 0.400 |
| Time constraint x Metal larval treatment | 1 | 0.118 | 0.731 |
| Egg treatment x Metal larval treatment | 1 | 0.000 | 0.999 |
| Time constraint x Predator larval treatment | 1 | 0.883 | 0.347 |
| Egg treatment x Predator larval treatment | 1 | 2.714 | 0.099 |
| Metal larval treatment x Predator larval treatment | 1 | 0.000 | 0.999 |
| Time constraint x Sex | 1 | 0.603 | 0.437 |
| Egg treatment x Sex | 1 | 0.000 | 0.999 |
| Metal larval treatment x Sex | 1 | 0.955 | 0.328 |
| Predator larval treatment x Sex | 1 | 0.000 | 0.999 |
| Time constraint x Egg treatment x Metal larval treatment | 1 | 0.000 | 0.999 |
| Time constraint x Egg treatment x Predator larval treatment | 1 | 1.726 | 0.189 |
| Time constraint x Metal larval treatment x Predator larval treatment | 1 | 0.000 | 1.000 |
| Egg treatment x Metal larval treatment x Predator larval treatment | 1 | 0.000 | 0.999 |
| Time constraint x Egg treatment x Sex | 1 | 0.000 | 0.999 |
| Time constraint x Metal larval treatment x Sex | 1 | 0.359 | 0.548 |
| Egg treatment x Metal larval treatment x Sex | 1 | 0.000 | 0.999 |
| Time constraint x Predator larval treatment x Sex | 1 | 0.000 | 1.000 |
| Egg treatment x Predator larval treatment x Sex | 1 | 0.000 | 0.999 |
| Metal larval treatment x Predator larval treatment x Sex | 1 | 0.000 | 0.999 |
| Time constraint x Egg treatment x Metal larval treatment x Predator larval treatment | 1 | 0.000 | 0.999 |
| Time constraint x Egg treatment x Metal larval treatment x Sex | 1 | 0.000 | 0.999 |
| Time constraint x Egg treatment x Predator larval treatment x Sex | 1 | 0.000 | 0.999 |
| Time constraint x Metal larval treatment x Predator larval treatment x Sex | 1 | 0.000 | 1.000 |
| Egg treatment x Metal larval treatment x Predator larval treatment x Sex | 1 | 0.000 | 0.999 |
| Time constraint x Egg treatment x Metal larval treatment x Predator larval treatment x Sex | 1 | 0.000 | 0.999 |
| **Egg development time** | | | |
| Egg treatment | 1 | 6.67e^-01^ | 0.678 |
| Time constraint | 1 | 1.53e^-05^ | 0.414 |
| Egg treatment x time constraint | 1 | 2.50e^02^ | **< 0.001** |
| **Mass at F-0** | | | |
| Egg treatment | 1 | 2.095 | 0.147 |
| Time constraint | 1 | 1.113 | 0.291 |
| Sex | 1 | 27.235 | **< 0.001** |
| Egg treatment x time constraint | 1 | 4.745 | **0.029^*^** |
| Egg treatment x sex | 1 | 1.068 | 0.301 |
| Time constraint x sex | 1 | 2.124 | 0.144 |
| Egg treatment x time constraint x sex | 1 | 0.680 | 0.409 |
| **Larval development time until F-0** | | | |
| Egg treatment | 1 | 1.661 | 0.197 |
| Time constraint | 1 | 16.417 | **< 0.001** |
| Sex | 1 | 0.763 | 0.382 |
| Egg treatment x time constraint | 1 | 1.020 | 0.088 |
| Egg treatment x sex | 1 | 0.967 | 0.325 |
| Time constraint x sex | 1 | 0.189 | 0.664 |
| Egg treatment x time constraint x sex | 1 | 1.010 | 0.164 |
| **Growth rate until F-0** | | | |
| Egg treatment | 1 | 2.058 | 0.151 |
| Time constraint | 1 | 14.546 | **0.000** |
| Sex | 1 | 37.670 | **< 0.001** |
| Egg treatment x time constraint | 1 | 9.726 | **0.002** |
| Egg treatment x sex | 1 | 5.984 | **0.014** |
| Time constraint x sex | 1 | 5.511 | **0.019** |
| Egg treatment x time constraint x sex | 1 | 3.961 | **0.047** |
| **Growth rate during larval treatment** | | | |
| Egg treatment | 1 | 3.076 | 0.079 |
| Time constraint | 1 | 35.314 | **< 0.001** |
| Metal larval treatment | 1 | 0.932 | 0.334 |
| Predator larval treatment | 1 | 2.602 | 0.106 |
| Sex | 1 | 0.902 | 0.342 |
| Egg treatment x Time constraint | 1 | 0.144 | 0.704 |
| Egg treatment x Predator larval treatment | 1 | 0.025 | 0.874 |
| Time constraint x Predator larval treatment | 1 | 0.932 | 0.334 |
| Egg treatment x Metal larval treatment | 1 | 2.063 | 0.150 |
| Time constraint x Metal larval treatment | 1 | 1.665 | 0.196 |
| Predator larval treatment x Metal larval treatment | 1 | 0.208 | 0.648 |
| Egg treatment x Sex | 1 | 0.189 | 0.664 |
| Time constraint x Sex | 1 | 0.470 | 0.492 |
| Predator larval treatment x Sex | 1 | 0.029 | 0.864 |
| Metal larval treatment x Sex | 1 | 1.283 | 0.257 |
| Egg treatment x Time constraint x Predator larval treatment | 1 | 0.378 | 0.538 |
| Egg treatment x Time constraint x Metal larval treatment | 1 | 0.109 | 0.741 |
| Egg treatment x Predator larval treatment x Metal larval treatment | 1 | 0.000 | 0.997 |
| Time constraint x Predator larval treatment x Metal larval treatment | 1 | 0.890 | 0.345 |
| Egg treatment x Time constraint x Sex | 1 | 5.159 | **0.02** |
| Egg treatment x Predator larval treatment x Sex | 1 | 0.624 | 0.429 |
| Time constraint x Predator larval treatment x Sex | 1 | 2.551 | 0.110 |
| Egg treatment x Metal larval treatment x Sex | 1 | 0.140 | 0.708 |
| Time constraint x Metal larval treatment x Sex | 1 | 0.013 | 0.908 |
| Predator larval treatment x Metal larval treatment x Sex | 1 | 0.819 | 0.365 |
| Egg treatment x Time constraint x Predator larval treatment x Metal larval treatment | 1 | 0.003 | 0.958 |
| Egg treatment x Time constraint x Predator larval treatment x Sex | 1 | 0.378 | 0.538 |
| Egg treatment x Time constraint x Metal larval treatment x Sex | 1 | 3.990 | 0.055 |
| Egg treatment x Predator larval treatment x Metal larval treatment x Sex | 1 | 0.093 | 0.759 |
| Time constraint x Predator larval treatment x Metal larval treatment x Sex | 1 | 0.043 | 0.835 |
| Egg treatment x Time constraint x Predator larval treatment x Metal larval treatment x Sex | 1 | 0.568 | 0.451 |
| **Behavioral traits** | | | |
| **Activity** | | | |
| Egg treatment | 1 | 2.231 | 0.135 |
| Time constraint | 1 | 3.449 | 0.063 |
| Metal larval treatment | 1 | 0.223 | 0.636 |
| Predator larval treatment | 1 | 0.410 | 0.512 |
| Sex | 1 | 1.055 | 0.304 |
| Egg treatment x Time constraint | 1 | 4.072 | **0.043** |
| Egg treatment x Metal larval treatment | 1 | 0.800 | 0.371 |
| Time constraint x Metal larval treatment | 1 | 0.346 | 0.556 |
| Egg treatment x Predator larval treatment | 1 | 0.884 | 0.347 |
| Time constraint x Predator larval treatment | 1 | 0.005 | 0.939 |
| Metal larval treatment x Predator larval treatment | 1 | 0.007 | 0.932 |
| Egg treatment x Sex | 1 | 0.339 | 0.559 |
| Time constraint x Sex | 1 | 1.543 | 0.214 |
| Metal larval treatment x Sex | 1 | 0.124 | 0.724 |
| Predator larval treatment x Sex | 1 | 0.000 | 0.995 |
| Egg treatment x Time constraint x Metal larval treatment | 1 | 0.150 | 0.698 |
| Egg treatment x Time constraint x Predator larval treatment | 1 | 0.135 | 0.713 |
| Egg treatment x Metal larval treatment x Predator larval treatment | 1 | 0.027 | 0.868 |
| Time constraint x Metal larval treatment x Predator larval treatment | 1 | 0.032 | 0.857 |
| Egg treatment x Time constraint x Sex | 1 | 0.6055 | 0.436 |
| Egg treatment x Metal larval treatment x Sex | 1 | 0.209 | 0.647 |
| Time constraint x Metal larval treatment x Sex | 1 | 0.165 | 0.684 |
| Egg treatment x Predator larval treatment x Sex | 1 | 2.736 | 0.098 |
| Time constraint x Predator larval treatment x Sex | 1 | 0.568 | 0.451 |
| Metal larval treatment x Predator larval treatment x Sex | 1 | 0.116 | 0.734 |
| Egg treatment x Time constraint x Metal larval treatment x Predator larval treatment | 1 | 0.693 | 0.405 |
| Egg treatment x Time constraint x Metal larval treatment x Sex | 1 | 0.739 | 0.389 |
| Egg treatment x Time constraint x Predator larval treatment x Sex | 1 | 5.954 | **0.015** |
| Egg treatment x Metal larval treatment x Predator larval treatment x Sex | 1 | 2.030 | 0.154 |
| Time constraint x Metal larval treatment x Predator larval treatment x Sex | 1 | 0.606 | 0.436 |
| Egg treatment x Time constraint x Metal larval treatment x Predator larval treatment x Sex | 1 | 3.918 | 0.057 |
| **Resting time** | | | |
| Egg treatment | 1 | 1.262 | 0.261 |
| Time constraint | 1 | 0.775 | 0.378 |
| Metal larval treatment | 1 | 0.004 | 0.949 |
| Predator larval treatment | 1 | 1.350 | 0.245 |
| Sex | 1 | 0.055 | 0.813 |
| Egg treatment x Time constraint | 1 | 0.185 | 0.666 |
| Egg treatment x Metal larval treatment | 1 | 1.823 | 0.176 |
| Time constraint x Metal larval treatment | 1 | 0.001 | 0.979 |
| Egg treatment x Predator larval treatment | 1 | 3.752 | 0.053 |
| Time constraint x Predator larval treatment | 1 | 0.027 | 0.867 |
| Metal larval treatment x Predator larval treatment | 1 | 0.009 | 0.923 |
| Egg treatment x Sex | 1 | 1.716 | 0.190 |
| Time constraint x Sex | 1 | 0.098 | 0.753 |
| Metal larval treatment x Sex | 1 | 1.558 | 0.211 |
| Predator larval treatment x Sex | 1 | 1.150 | 0.283 |
| Egg treatment x Time constraint x Metal larval treatment | 1 | 0.172 | 0.678 |
| Egg treatment x Time constraint x Predator larval treatment | 1 | 0.713 | 0.398 |
| Egg treatment x Metal larval treatment x Predator larval treatment | 1 | 0.448 | 0.503 |
| Time constraint x Metal larval treatment x Predator larval treatment | 1 | 0.014 | 0.905 |
| Egg treatment x Time constraint x Sex | 1 | 1.134 | 0.287 |
| Egg treatment x Metal larval treatment x Sex | 1 | 0.153 | 0.695 |
| Time constraint x Metal larval treatment x Sex | 1 | 1.403 | 0.236 |
| Egg treatment x Predator larval treatment x Sex | 1 | 2.729 | 0.098 |
| Time constraint x Predator larval treatment x Sex | 1 | 0.229 | 0.632 |
| Metal larval treatment x Predator larval treatment x Sex | 1 | 0.447 | 0.503 |
| Egg treatment x Time constraint x Metal larval treatment x Predator larval treatment | 1 | 0.004 | 0.952 |
| Egg treatment x Time constraint x Metal larval treatment x Sex | 1 | 0.002 | 0.962 |
| Egg treatment x Time constraint x Predator larval treatment x Sex | 1 | 2.849 | 0.091 |
| Egg treatment x Metal larval treatment x Predator larval treatment x Sex | 1 | 0.213 | 0.645 |
| Time constraint x Metal larval treatment x Predator larval treatment x Sex | 1 | 0.496 | 0.481 |
| Egg treatment x Time constraint x Metal larval treatment x Predator larval treatment x Sex | 1 | 0.039 | 0.842 |
| **Freezing time** | | | |
| Egg treatment | 1 | 0.393 | 0.530 |
| Time constraint | 1 | 0.083 | 0.773 |
| Metal larval treatment | 1 | 1.062 | 0.303 |
| Predator larval treatment | 1 | 0.152 | 0.696 |
| Sex | 1 | 0.545 | 0.460 |
| Egg treatment x Time constraint | 1 | 0.044 | 0.834 |
| Egg treatment x Metal larval treatment | 1 | 0.302 | 0.582 |
| Time constraint x Metal larval treatment | 1 | 0.050 | 0.822 |
| Egg treatment x Predator larval treatment | 1 | 0.841 | 0.359 |
| Time constraint x Predator larval treatment | 1 | 0.018 | 0.893 |
| Metal larval treatment x Predator larval treatment | 1 | 0.762 | 0.383 |
| Egg treatment x Sex | 1 | 0.935 | 0.334 |
| Time constraint x Sex | 1 | 0.003 | 0.957 |
| Metal larval treatment x Sex | 1 | 0.120 | 0.290 |
| Predator larval treatment x Sex | 1 | 0.978 | 0.323 |
| Egg treatment x Time constraint x Metal larval treatment | 1 | 0.043 | 0.836 |
| Egg treatment x Time constraint x Predator larval treatment | 1 | 0.089 | 0.765 |
| Egg treatment x Metal larval treatment x Predator larval treatment | 1 | 0.748 | 0.388 |
| Time constraint x Metal larval treatment x Predator larval treatment | 1 | 0.239 | 0.625 |
| Egg treatment x Time constraint x Sex | 1 | 0.116 | 0.732 |
| Egg treatment x Metal larval treatment x Sex | 1 | 0.509 | 0.475 |
| Time constraint x Metal larval treatment x Sex | 1 | 0.183 | 0.669 |
| Egg treatment x Predator larval treatment x Sex | 1 | 3.423 | 0.064 |
| Time constraint x Predator larval treatment x Sex | 1 | 0.108 | 0.742 |
| Metal larval treatment x Predator larval treatment x Sex | 1 | 2.109 | 0.146 |
| Egg treatment x Time constraint x Metal larval treatment x Predator larval treatment | 1 | 0.509 | 0.475 |
| Egg treatment x Time constraint x Metal larval treatment x Sex | 1 | 0.172 | 0.678 |
| Egg treatment x Time constraint x Predator larval treatment x Sex | 1 | 1.239 | 0.265 |
| Egg treatment x Metal larval treatment x Predator larval treatment x Sex | 1 | 2.744 | 0.097 |
| Time constraint x Metal larval treatment x Predator larval treatment x Sex | 1 | 0.875 | 0.349 |
| Egg treatment x Time constraint x Metal larval treatment x Predator larval treatment x Sex | 1 | 1.375 | 0.241 |
| **Feeding rate** | | | |
| Egg treatment | 1 | 0.444 | 0.505 |
| Time constraint | 1 | 0.003 | 0.956 |
| Predator larval treatment | 1 | 8.491 | **0.003** |
| Metal larval treatment | 1 | 5.012 | 0.055 |
| Sex | 1 | 5.122 | 0.07 |
| Egg treatment x Time constraint | 1 | 0.171 | 0.679 |
| Egg treatment x Predator larval treatment | 1 | 0.581 | 0.446 |
| Time constraint x Predator larval treatment | 1 | 2.464 | 0.116 |
| Egg treatment x Metal larval treatment | 1 | 0.136 | 0.712 |
| Time constraint x Metal larval treatment | 1 | 4.363 | 0.086 |
| Predator larval treatment x Metal larval treatment | 1 | 4.956 | 0.088 |
| Egg treatment x Sex | 1 | 3.152 | 0.075 |
| Time constraint x Sex | 1 | 7.270 | 0.007 |
| Predator larval treatment x Sex | 1 | 5.065 | **0.024** |
| Metal larval treatment x Sex | 1 | 4.636 | 0.071 |
| Egg treatment x Time constraint x Predator larval treatment | 1 | 0.479 | 0.488 |
| Egg treatment x Time constraint x Metal larval treatment | 1 | 0.021 | 0.883 |
| Egg treatment x Predator larval treatment x Metal larval treatment | 1 | 1.302 | 0.253 |
| Time constraint x Predator larval treatment x Metal larval treatment | 1 | 3.962 | 0.086 |
| Egg treatment x Time constraint x Sex | 1 | 2.372 | 0.123 |
| Egg treatment x Predator larval treatment x Sex | 1 | 1.140 | 0.285 |
| Time constraint x Predator larval treatment x Sex | 1 | 8.481 | **0.003** |
| Egg treatment x Metal larval treatment x Sex | 1 | 0.007 | 0.930 |
| Time constraint x Metal larval treatment x Sex | 1 | 1.261 | 0.101 |
| Predator larval treatment x Metal larval treatment x Sex | 1 | 5.084 | 0.057 |
| Egg treatment x Time constraint x Predator larval treatment x Metal larval treatment | 1 | 0.611 | 0.434 |
| Egg treatment x Time constraint x Predator larval treatment x Sex | 1 | 4.945 | **0.049** |
| Egg treatment x Time constraint x Metal larval treatment x Sex | 1 | 1.418 | 0.233 |
| Egg treatment x Predator larval treatment x Metal larval treatment x Sex | 1 | 2.771 | 0.095 |
| Time constraint x Predator larval treatment x Metal larval treatment x Sex | 1 | 8.192 | 0.054 |
| Egg treatment x Time constraint x Predator larval treatment x Metal larval treatment x Sex | 1 | 3.707 | 0.096 |
| **Physiology Traits** | | | |
| **MDA** |  |  |  |
| Egg treatment | 1 | 0.314 | 0.575 |
| Time constraint | 1 | 4.706 | **0.030** |
| Metal larval treatment | 1 | 0.090 | 0.764 |
| Predator larval treatment | 1 | 0.061 | 0.804 |
| Sex | 1 | 0.268 | 0.604 |
| Egg treatment x Time_constraint | 1 | 0.273 | 0.601 |
| Egg treatment x Metal larval treatment | 1 | 0.005 | 0.941 |
| Time constraint x Metal larval treatment | 1 | 0.174 | 0.676 |
| Egg treatment x Predator larval treatment | 1 | 0.001 | 0.980 |
| Time constraint x Predator larval treatment | 1 | 1.313 | 0.252 |
| Metal larval treatment x Predator larval treatment | 1 | 0.045 | 0.832 |
| Egg treatment x Sex | 1 | 0.055 | 0.814 |
| Time constraint x Sex | 1 | 0.279 | 0.597 |
| Metal larval treatment x Sex | 1 | 0.000 | 0.998 |
| Predator larval treatment x Sex | 1 | 0.006 | 0.937 |
| Egg treatment x Time constraint x Metal larval treatment | 1 | 0.044 | 0.834 |
| Egg treatment x Time constraint x Predator larval treatment | 1 | 0.005 | 0.943 |
| Egg treatment x Metal larval treatment x Predator larval treatment | 1 | 0.006 | 0.937 |
| Time constraint x Metal larval treatment x Predator larval treatment | 1 | 0.637 | 0.425 |
| Egg treatment x Time constraint x Sex | 1 | 0.0643 | 0.799 |
| Egg treatment x Metal larval treatment x Sex | 1 | 0.009 | 0.923 |
| Time constraint x Metal larval treatment x Sex | 1 | 0.807 | 0.369 |
| Egg treatment x Predator larval treatment x Sex | 1 | 0.005 | 0.941 |
| Time constraint x Predator larval treatment x Sex | 1 | 0.286 | 0.592 |
| Metal larval treatment x Predator larval treatment x Sex | 1 | 1.496 | 0.221 |
| Egg treatment x Time constraint x Metal larval treatment x Predator larval treatment | 1 | 0.239 | 0.625 |
| Egg treatment x Time constraint x Metal larval treatment x Sex | 1 | 0.728 | 0.394 |
| Egg treatment x Time constraint x Predator larval treatment x Sex | 1 | 0.030 | 0.861 |
| Egg treatment x Metal larval treatment x Predator larval treatment x Sex | 1 | 0.073 | 0.787 |
| Time constraint x Metal larval treatment x Predator larval treatment x Sex | 1 | 0.004 | 0.951 |
| Egg treatment x Time constraint x Metal larval treatment x Predator larval treatment x Sex | 1 | 0.419 | 0.518 |
| **CEA** | | | |
| Egg treatment | 1 | 2.942 | 0.086 |
| Time constraint | 1 | 1.126 | 0.288 |
| Metal larval treatment | 1 | 1.827 | 0.176 |
| Predator larval treatment | 1 | 0.613 | 0.433 |
| Sex | 1 | 3.279 | 0.070 |
| Egg treatment x Time constraint | 1 | 5.66 | **0.017** |
| Egg treatment x Metal larval treatment | 1 | 0.007 | 0.934 |
| Time constraint x Metal larval treatment | 1 | 0.467 | 0.494 |
| Egg treatment x Predator larval treatment | 1 | 0.193 | 0.661 |
| Time constraint x Predator larval treatment | 1 | 0.130 | 0.718 |
| Metal larval treatment x Predator larval treatment | 1 | 2.251 | 0.133 |
| Egg treatment x Sex | 1 | 0.756 | 0.384 |
| Time constraint x Sex | 1 | 0.271 | 0.602 |
| Metal larval treatment x Sex | 1 | 2.349 | 0.051 |
| Predator larval treatment x Sex | 1 | 5.988 | **0.014** |
| Egg treatment x Time constraint x Metal larval treatment | 1 | 0.096 | 0.756 |
| Egg treatment x Time constraint x Predator larval treatment | 1 | 0.231 | 0.631 |
| Egg treatment x Metal larval treatment x Predator larval treatment | 1 | 0.417 | 0.518 |
| Time constraint x Metal larval treatment x Predator larval treatment | 1 | 0.082 | 0.774 |
| Egg treatment x Time constraint x Sex | 1 | 0.074 | 0.786 |
| Egg treatment x Metal larval treatment x Sex | 1 | 0.575 | 0.448 |
| Time constraint x Metal larval treatment x Sex | 1 | 0.528 | 0.467 |
| Time constraint x Metal larval treatment x Sex | 1 | 0.528 | 0.467 |
| Egg treatment x Predator larval treatment x Sex | 1 | 1.276 | 0.258 |
| Time constraint x Predator larval treatment x Sex | 1 | 1.129 | 0.288 |
| Metal larval treatment x Predator larval treatment x Sex | 1 | 5.286 | **0.021** |
| Egg treatment x Time constraint x Metal larval treatment x Predator larval treatment | 1 | 0.017 | 0.895 |
| Egg treatment x Time constraint x Metal larval treatment x Sex | 1 | 0.019 | 0.889 |
| Egg treatment x Time constraint x Predator larval treatment x Sex | 1 | 0.524 | 0.468 |
| Egg treatment x Metal larval treatment x Predator larval treatment x Sex | 1 | 1.451 | 0.229 |
| Time constraint x Metal larval treatment x Predator larval treatment x Sex | 1 | 0.685 | 0.407 |
| Egg treatment x Time constraint x Metal larval treatment x Predator larval treatment x Sex | 1 | 0.483 | 0.486 |

**Table S5**. Results of the model selection analysis for life history traits (survival 14 days after hatching, survival at F0, survival after 5 days larval treatment, mass at F0, egg development time, development time (hatching to F-0), growth rate at F0, growth rate during the 5 days larval treatment), behavioural traits (activity, freezing time, boldness, feeding rate), and physiological traits (MDA and CEA). For each variable, we initially fitted a full GLM model including all fixed effects and their interactions. Then, we ran a model selection analysis based on the Akaike’s information criteria for sample size (AICc) and weight as criteria to determine the best explanatory model (indicated in red). The final model was refitted using only the variables retained in the best explanatory model.

| **Life history traits** | | | | | | | | |
| --- | --- | --- | --- | --- | --- | --- | --- | --- |
| **Model** | **df** | | **logLik** | **AICc** | | **delta** | **weight** | |
| **Survival 14-days after hatching** | | | | | | | | |
| Time constraint +Egg treatment+ Time constraint:Egg treatment | 5 | | -388.31 | 786.69 | | 0.00 | 1 | |
| Time constraint +Egg treatment | 4 | | -400.03 | 808.12 | | 21.43 | 0 | |
| Time constraint | 3 | | -402.48 | 810.99 | | 24.29 | 0 | |
| Egg treatment | 3 | | -415.70 | 837.44 | | 50.74 | 0 | |
| **Survival at F-0** | | | | | | | | |
| Time constraint | 2 | | -347.89 | 699.79 | | 0.00 | 0.44 | |
| Time constraint +Egg treatment+ Time constraint:Egg treatment | 4 | | -346.39 | 700.86 | | 0.59 | 0.33 | |
| Time constraint +Egg treatment | 3 | | -347.78 | 701.61 | | 2.36 | 0.14 | |
| Null | 1 | | -350.40 | 702.82 | | 3.03 | 0.10 | |
| **Survival after larval treatment** | | | | | | | | |
| Time constraint+Metal larval-treatment | 3 | | -97.40 | 200.87 | | 0.00 | 0.30 | |
| Time constraint | 2 | | -98.52 | 201.08 | | 0.21 | 0.27 | |
| Time constraint+Egg treatment+Metal larval-treatment | 4 | | -96.68 | 201.46 | | 0.59 | 0.22 | |
| Time constraint+Egg treatment | 3 | | -97.80 | 201.66 | | 0.79 | 0.20 | |
| **Egg development time** | | | | | | | | |
| Time constraint+Egg treatment+Time constraint:Egg treatment | 5 | | 14.29 | -18.47 | | 0.00 | 1 | |
| Time constraint+Egg treatment | 4 | | -89.56 | 187.19 | | 205.67 | 0 | |
| Time constraint | 3 | | -133.50 | 273.05 | | 291.53 | 0 | |
| Egg treatment | 3 | | -144.19 | 294.43 | | 312.90 | 0 | |
| **Growth rate until F-0** | | | | | | | | |
| Time constraint+egg treatment+sex+Time constraint:Egg treatment+Time constraint:Sex+Egg treatment:Sex+Time constraint:Egg treatment:Sex | 9 | | 193.71 | -368.90 | | 0.00 | 0.66 | |
| Time constraint+egg treatment+sex+Time constraint:egg treatment+Time constraint:sex+egg treatment:sex | 8 | | 191.69 | -366.98 | | 1.93 | 0.25 | |
| Time constraint+egg treatment+sex+Time constraint:egg treatment+egg treatment:sex | 7 | | 188.93 | -363.54 | | 5.36 | 0.05 | |
| Time constraint+egg treatment+sex+Time constraint:egg treatment+Time constraint:sex | 7 | | 188.69 | -363.07 | | 5.84 | 0.04 | |
| **Growth rate during larval treatment** | | | | | | | | |
| Time constraint+Egg treatment+Predator larval-treatment | 5 | | -410.84 | 831.87 | | 0.00 | 0.38 | |
| Time constraint+Predator larval-treatment | 4 | | -412.32 | 832.75 | | 0.88 | 0.25 | |
| Time constraint+Egg treatment | 4 | | -412.40 | 832.93 | | 1.06 | 0.23 | |
| Time constraint | 3 | | -413.86 | 833.78 | | 1.91 | 0.15 | |
|  | | | | | | | | |
| **Behavioral traits** | | | | | | | | |
| **Activity** | | | | | | | | |
| Time constraint+Egg treatment+Predator larval-treatment+Time constraint:Egg treatment+Egg treatment:Predator larval treatment | | 7 | -309.80 | | 634.09 | 0.00 | | 0.44 |
| Time constraint+egg treatment+predator larval-treatment+Time constraint:egg treatment+Egg_treatment:Predator larval-treatment+Time constraint: Predator_larval-treatment | | 8 | -309.47 | | 635.56 | 1.48 | | 0.21 |
| Time constraint+egg treatment+predator larval- treatment+Time constraint:egg treatment+Egg_treatment:Predator larval-treatment+Time constraint: Predator_larval-treatment+ Time constraint: egg treatment:Predator_larval-treatment | | 9 | -308.58 | | 635.94 | 1.85 | | 0.18 |
| Time constraint+egg treatment+predator larval-treatment+Metal larval-treatment+ Egg_treatment:Predator_larval-treatment+Time constraint: egg treatment | | 8 | -309.67 | | 635.97 | 1.88 | | 0.17 |
| **Resting time** | | | | | | | | |
| Time constraint+Egg treatment+Time constraint:Egg treatment | | 5 | -1339.62 | | 2689.49 | 0.00 | | 0.29 |
| Time constraint+egg treatment+Metal larval-treatment+ Egg_treatment:Metal_larval-treatment+Time_constraint: Egg_treatment | | 7 | -1337.63 | | 2689.74 | 0.25 | | 0.26 |
| Time constraint+egg treatment+Predator larval-treatment+ Egg_treatment:Predator_larval-treatment+Time_constraint: Egg_treatment | | 7 | -1337.74 | | 2689.97 | 0.48 | | 0.23 |
| Time constraint+egg treatment+Metal larval-treatment+Predator larval-treatment+ Egg_treatment:Metal larval-treatment+Egg_treatment:Predator larval-treatment+ Time_constraint: Egg_treatment | | 9 | -1335.65 | | 2690.08 | 0.59 | | 0.22 |
| **Freezing time** | | | | | | | | |
| Null | | 2 | -444.59 | | 893.22 | 0.00 | | 0.30 |
| Metal larval-treatment | | 3 | -443.66 | | 893.41 | 0.19 | | 0.27 |
| Predator larval-treatment | | 3 | -443.87 | | 893.83 | 0.61 | | 0.22 |
| Metal larval-treatment+Predator larval-treatment | | 4 | -442.91 | | 893.95 | 0.73 | | 0.21 |
| **Feeding rate** | | | | | | | | |
| Time constraint+Predator larval treatment | | 4 | -1005.19 | | 2018.51 | 0.00 | | 0.39 |
| Time constraint+egg treatment+predator larval-treatment | | 5 | -1004.54 | | 2019.29 | 0.78 | | 0.27 |
| Time constraint+egg treatment+predator larval-treatment+Time_constraint:egg treatment | | 6 | -1003.85 | | 2019.99 | 1.48 | | 0.19 |
| Time constraint+predator larval-treatment+Time constraint:predator larval-treatment | | 5 | -1005.08 | | 2020.37 | 1.86 | | 0.15 |
|  | | | | | | | | |
| **Physiology Traits** | | | | | | | | |
| **MDA** | | | | | | | | |
| Time constraint+Metal larval-treatment+Predator larval treatment+Time constraint: Metal larval-treatment | | 6 | -162.33 | | 336.93 | 0.00 | | 0.27 |
| Time constraint+Metal larval-treatment+Predator larval-treatment+Time constraint: Metal larval-treatment+Time constraint: predator larval-treatment | | 7 | -161.29 | | 336.93 | 0.00 | | 0.27 |
| Time constraint+Metal larval-treatment+Predator larval-treatment+Time constraint: Metal larval-treatment+Time constraint: predator larval-treatment+ Time_constraint:egg treatment | | 9 | -159.33 | | 337.22 | 0.29 | | 0.23 |
| Time constraint+Egg treatment+Metal larval-treatment+Predator larval-treatment+Time constraint: Metal larval-treatment+Time_constraint:egg treatment | | 8 | -160.41 | | 337.26 | 0.34 | | 0.23 |
| **CEA** | | | | | | | | |
| Time constraint+Egg treatment+Predator larval treatment+Time constraint:Egg treatment+Time constraint:Predator larval treatment | | 7 | -11.70 | | 37.74 | 0.00 | | 0.47 |
| Time constraint+egg treatment+Time constraint: egg treatment | | 5 | -14.17 | | 38.53 | 0.79 | | 0.32 |
| Time constraint+egg treatment+predator larval-treatment+Time constraint: egg treatment | | 6 | -13.58 | | 39.43 | 1.68 | | 0.20 |
| Null | | 2 | -25.02 | | 54.08 | 16.34 | | 0.00 |

**Table S6** Multiple comparisons of means using Tukey’s HSD contrasts for life history traits up to F-0 stage (egg development time, survival 14 days after hatching, survival until F-0 and growth rate until F-0), after 5 days larval treatments (Survival after larval treatment and growth rate during larval treatment), behavior traits (activity, resting time, and feeding rate), and physiological traits (MDA and CEA) under the effect of egg treatment (no predator and predator cues), time constraint (Weak and Strong), metal larval treatment (metal no/yes), and predator larval treatment (predator no/yes). Significant p-values are in bold: p < 0.05.

| **Contrast** | **Estimate** | **SE** | **Df** | **t ratio** | **p value** |
| --- | --- | --- | --- | --- | --- |
| **Egg development time** | | | | | |
| Weak No predator - Strong No predator | -0.0111 | 0.0267 | 543 | -0.415 | 0.9758 |
| Weak No predator - Weak Predator cue | 0.0219 | 0.0268 | 543 | 0.817 | 0.8463 |
| Weak No predator - Strong Predator cue | -0.6637 | 0.0333 | 543 | -19.942 | **<0.0001** |
| Strong No predator - Weak Predator cue | 0.0330 | 0.0266 | 543 | 1.240 | 0.6014 |
| Strong No predator - Strong Predator cue | -0.6526 | 0.0331 | 543 | -19.710 | **<0.0001** |
| Weak Predator cue - Strong Predator cue | -0.6856 | 0.0332 | 543 | -20.643 | **<0.0001** |
| **Survival 14 days after hatching** | | | | | |
| No predator Strong - Predator cue Strong | 1.535 | 0.290 | Inf | 5.289 | **<0.0001** |
| No predator Strong - No predator Weak | -0.363 | 0.309 | Inf | -1.175 | 0.6430 |
| No predator Strong - Predator cue Weak | -1.155 | 0.347 | Inf | -3.325 | **0.0049** |
| Predator cue Strong - No predator Weak | -1.897 | 0.304 | Inf | -6.251 | **<0.0001** |
| Predator cue Strong - Predator cue Weak | -2.689 | 0.343 | Inf | -7.832 | **<0.0001** |
| No predator Weak - Predator cue Weak | -0.792 | 0355 | Inf | -2.228 | **0.0115** |
| **Survival until F-0** | | | | | |
| Weak - Strong | 0.408 | 0.182 | Inf | 2.244 | **0.0248** |
| **Growth rate until F-0** | | | | | |
| No predator Weak Female - Predator cue Weak Female | -0.0726 | 0.0259 | 354 | -2.806 | 0.0967 |
| No predator Weak Female - No predator Strong Female | -0.1092 | 0.0288 | 354 | -3.791 | **0.0043** |
| No predator Weak Female - Predator cue Strong Female | -0.1359 | 0.0345 | 354 | -3.936 | **0.0025** |
| No predator Weak Female - No predator Weak Male | 0.0553 | 0.0284 | 354 | 1.948 | 0.5186 |
| No predator Weak Female - Predator cue Weak Male | 0.0129 | 0.0305 | 354 | 0.423 | 0.9999 |
| No predator Weak Female - No predator Strong Male | -0.0273 | 0.0281 | 354 | -0.973 | 0.9779 |
| No predator Weak Female - Predator cue Strong Male | 0.1084 | 0.0401 | 354 | 2.701 | 0.1258 |
| Predator cue Weak Female - No predator Strong Female | -0.0366 | 0.0264 | 354 | -1.390 | 0.8614 |
| Predator cue Weak Female - Predator cue Strong Female | -0.0633 | 0.0325 | 354 | -1.948 | 0.5191 |
| Predator cue Weak Female - No predator Weak Male | 0.1279 | 0.0259 | 354 | 4.943 | **<0.0001** |
| Predator cue Weak Female - Predator cue Weak Male | 0.0855 | 0.0282 | 354 | 3.035 | 0.0520 |
| Predator cue Weak Female - No predator Strong Male | 0.0453 | 0.0256 | 354 | 1.769 | 0.6412 |
| Predator cue Weak Female - Predator cue Strong Male | 0.1810 | 0.0384 | 354 | 4.713 | **<0.0001** |
| No predator Strong Female - Predator cue Strong Female | -0.0267 | 0.0349 | 354 | -0.764 | 0.9948 |
| No predator Strong Female - No predator Weak Male | 0.1645 | 0.0288 | 354 | 5.709 | **<0.0001** |
| No predator Strong Female - Predator cue Weak Male | 0.1221 | 0.0309 | 354 | 3.953 | **0.0023** |
| No predator Strong Female - No predator Strong Male | 0.0819 | 0.0286 | 354 | 2.868 | 0.0823 |
| No predator Strong Female - Predator cue Strong Male | 0.2176 | 0.0404 | 354 | 5.381 | **<0.0001** |
| Predator cue Strong Female - No predator Weak Male | 0.1912 | 0.0345 | 354 | 5.537 | **<0.0001** |
| Predator cue Strong Female - Predator cue Weak Male | 0.1488 | 0.0363 | 354 | 4.101 | **0.0013** |
| Predator cue Strong Female - No predator Strong Male | 0.1086 | 0.0343 | 354 | 3.164 | **0.0357** |
| Predator cue Strong Female - Predator cue Strong Male | 0.2443 | 0.0447 | 354 | 5.466 | **<0.0001** |
| No predator Weak Male - Predator cue Weak Male | -0.0424 | 0.0305 | 354 | -1.391 | 0.8610 |
| No predator Weak Male - No predator Strong Male | -0.0826 | 0.0281 | 354 | -2.940 | 0.0679 |
| No predator Weak Male - Predator cue Strong Male | 0.0531 | 0.0401 | 354 | 1.323 | 0.8898 |
| Predator cue Weak Male - No predator Strong Male | -0.0402 | 0.0302 | 354 | -1.331 | 0.8866 |
| Predator cue Weak Male - Predator cue Strong Male | 0.0955 | 0.0416 | 354 | 2.293 | 0.3003 |
| No predator Strong Male - Predator cue Strong Male | 0.1357 | 0.0399 | 354 | 3.398 | **0.0171** |
| No predator Strong - Predator cue Strong | 0.0545 | 0.0265 | 354 | 2.056 | 0.1697 |
| No predator Strong - No predator Weak | 0.0959 | 0.0201 | 354 | 4.767 | **<0.0001** |
| No predator Strong - Predator cue Weak | 0.0384 | 0.0201 | 354 | 1.917 | 0.2229 |
| Predator cue Strong - No predator Weak | 0.0414 | 0.0265 | 354 | 1.564 | 0.4005 |
| Predator cue Strong - Predator cue Weak | -0.0161 | 0.0264 | 354 | -0.609 | 0.9291 |
| No predator Weak - Predator cue Weak | 0.0575 | 0.0200 | 354 | -2.877 | **0.0221** |
| **Survival after larval treatment** | | | | | |
| Weak - Strong | -0.993 | 0.472 | Inf | -2.086 | **0.0370** |
| Weak Metal Yes - Strong Metal Yes | -0.986 | 0.473 | Inf | -2.086 | 0.1577 |
| Weak Metal Yes - Weak Metal No | 0.591 | 0.401 | Inf | 1.475 | 0.4529 |
| Weak Metal Yes - Strong Metal No | -0.395 | 0.620 | Inf | -0.637 | 0.9199 |
| Strong Metal Yes - Weak Metal No | 1.577 | 0.619 | Inf | 2.547 | 0.0529 |
| Strong Metal Yes - Strong Metal No | 0.591 | 0.401 | Inf | 1.475 | 0.4529 |
| Weak Metal No - Strong Metal No | 0.986 | 0.473 | Inf | -2.086 | 0.1577 |
| **Growth rate during larval treatment** | | | | | |
| No predator Strong predator No - Predator cue Strong predator No | -0.16514 | 0.0966 | 327 | -1.710 | 0.6808 |
| No predator Strong predator No - No predator Weak predator No | 0.58093 | 0.0969 | 327 | 5.995 | **<.0001** |
| No predator Strong predator No - Predator cue Weak predator No | 0.41579 | 0.1190 | 327 | 3.481 | **0.0131** |
| No predator Strong predator No - No predator Strong predator Yes | -0.16303 | 0.0927 | 327 | -1.760 | 0.6478 |
| No predator Strong predator No - Predator cue Strong predator Yes | -0.32817 | 0.1340 | 327 | -2.450 | 0.2214 |
| No predator Strong predator No - No predator Weak predator Yes | 0.41790 | 0.1360 | 327 | 3.084 | **0.0454** |
| No predator Strong predator No - Predator cue Weak predator Yes | 0.25276 | 0.1530 | 327 | 1.657 | 0.7151 |
| Predator cue Strong predator No - No predator Weak predator No | 0.74607 | 0.1520 | 327 | 4.902 | **<.0001** |
| Predator cue Strong predator No - Predator cue Weak predator No | 0.58093 | 0.0969 | 327 | 5.995 | **<.0001** |
| Predator cue Strong predator No - No predator Strong predator Yes | 0.00211 | 0.1340 | 327 | 0.016 | 1.0000 |
| Predator cue Strong predator No - Predator cue Strong predator Yes | -0.16303 | 0.0927 | 327 | -1.760 | 0.6478 |
| Predator cue Strong predator No - No predator Weak predator Yes | 0.58304 | 0.1790 | 327 | 3.254 | **0.0272** |
| Predator cue Strong predator No - Predator cue Weak predator Yes | 0.41790 | 0.1360 | 327 | 3.084 | **0.0454** |
| No predator Weak predator No - Predator cue Weak predator No | -0.16514 | 0.0966 | 327 | -1.710 | 0.6808 |
| No predator Weak predator No - No predator Strong predator Yes | -0.74396 | 0.1330 | 327 | -5.610 | **<.0001** |
| No predator Weak predator No - Predator cue Strong predator Yes | -0.90910 | 0.1770 | 327 | -5.130 | **<.0001** |
| No predator Weak predator No - No predator Weak predator Yes | -0.16303 | 0.0927 | 327 | -1.760 | 0.6478 |
| No predator Weak predator No - Predator cue Weak predator Yes | -0.32817 | 0.1340 | 327 | -2.450 | 0.2214 |
| Predator cue Weak predator No - No predator Strong predator Yes | -0.57883 | 0.1500 | 327 | -3.865 | **0.0033** |
| Predator cue Weak predator No - Predator cue Strong predator Yes | -0.74396 | 0.1330 | 327 | -5.610 | **<.0001** |
| Predator cue Weak predator No - No predator Weak predator Yes | 0.00211 | 0.1340 | 327 | 0.016 | 1.0000 |
| Predator cue Weak predator No - Predator cue Weak predator Yes | -0.16303 | 0.0927 | 327 | -1.760 | 0.6478 |
| No predator Strong predator Yes - Predator cue Strong predator Yes | -0.16514 | 0.0966 | 327 | -1.710 | 0.6808 |
| No predator Strong predator Yes - No predator Weak predator Yes | 0.58093 | 0.0969 | 327 | 5.995 | **<.0001** |
| No predator Strong predator Yes - Predator cue Weak predator Yes | 0.41579 | 0.1190 | 327 | 3.481 | **0.0131** |
| Predator cue Strong predator Yes - No predator Weak predator Yes | 0.74607 | 0.1520 | 327 | 4.902 | **<.0001** |
| Predator cue Strong predator Yes - Predator cue Weak predator Yes | 0.58093 | 0.0969 | 327 | 5.995 | **<.0001** |
| No predator Weak predator Yes - Predator cue Weak predator Yes | -0.16514 | 0.0966 | 327 | -1.710 | 0.6808 |
| **Activity** | | | | | |
| No predator Strong Predator No - Predator cue Strong Predator No | 0.1237 | 0.214 | 236 | 0.578 | 0.9991 |
| No predator Strong Predator No - No predator Weak Predator No | 0.3303 | 0.148 | 236 | 2.237 | 0.3334 |
| No predator Strong Predator No - Predator cue Weak Predator No | -0.2480 | 0.195 | 236 | -1.270 | 0.9091 |
| No predator Strong Predator No - No predator Strong Predator Yes | -0.3995 | 0.148 | 236 | -2.706 | 0.1258 |
| No predator Strong Predator No - Predator cue Strong Predator Yes | 0.4766 | 0.218 | 236 | 2.186 | 0.3640 |
| No predator Strong Predator No - No predator Weak Predator Yes | -0.0692 | 0.214 | 236 | -0.323 | 1.0000 |
| No predator Strong Predator No - Predator cue Weak Predator Yes | 0.1049 | 0.191 | 236 | 0.548 | 0.9994 |
| Predator cue Strong Predator No - No predator Weak Predator No | 0.2066 | 0.210 | 236 | 0.982 | 0.9766 |
| Predator cue Strong Predator No - Predator cue Weak Predator No | -0.3717 | 0.184 | 236 | -2.016 | 0.4734 |
| Predator cue Strong Predator No - No predator Strong Predator Yes | -0.5231 | 0.211 | 236 | -2.479 | 0.2097 |
| Predator cue Strong Predator No - Predator cue Strong Predator Yes | 0.3529 | 0.177 | 236 | 1.989 | 0.4913 |
| Predator cue Strong Predator No - No predator Weak Predator Yes | -0.1928 | 0.212 | 236 | -0.908 | 0.9851 |
| Predator cue Strong Predator No - Predator cue Weak Predator Yes | -0.0188 | 0.249 | 236 | -0.075 | 1.0000 |
| No predator Weak Predator No - Predator cue Weak Predator No | -0.5783 | 0.191 | 236 | -3.022 | 0.0554 |
| No predator Weak Predator No - No predator Strong Predator Yes | -0.7297 | 0.204 | 236 | -3.585 | **0.0096** |
| No predator Weak Predator No - Predator cue Strong Predator Yes | 0.1463 | 0.214 | 236 | 0.682 | 0.9974 |
| No predator Weak Predator No - No predator Weak Predator Yes | -0.3995 | 0.148 | 236 | -2.706 | 0.1258 |
| No predator Weak Predator No - Predator cue Weak Predator Yes | -0.2254 | 0.187 | 236 | -1.203 | 0.9305 |
| Predator cue Weak Predator No - No predator Strong Predator Yes | -0.1514 | 0.192 | 236 | -0.788 | 0.9936 |
| Predator cue Weak Predator No - Predator cue Strong Predator Yes | 0.7246 | 0.262 | 236 | 2.764 | 0.1092 |
| Predator cue Weak Predator No - No predator Weak Predator Yes | 0.1789 | 0.194 | 236 | 0.923 | 0.9836 |
| Predator cue Weak Predator No - Predator cue Weak Predator Yes | 0.3529 | 0.177 | 236 | 1.989 | 0.4913 |
| No predator Strong Predator Yes - Predator cue Strong Predator Yes | 0.8761 | 0.215 | 236 | 4.072 | **0.0016** |
| No predator Strong Predator Yes - No predator Weak Predator Yes | 0.3303 | 0.148 | 236 | 2.237 | 0.3334 |
| No predator Strong Predator Yes - Predator cue Weak Predator Yes | 0.5044 | 0.188 | 236 | 2.680 | 0.1338 |
| Predator cue Strong Predator Yes - No predator Weak Predator Yes | -0.5458 | 0.217 | 236 | -2.520 | 0.1921 |
| Predator cue Strong Predator Yes - Predator cue Weak Predator Yes | -0.3717 | 0.184 | 236 | -2.016 | 0.4734 |
| No predator Weak Predator Yes - Predator cue Weak Predator Yes | 0.1741 | 0.190 | 236 | 0.917 | 0.9842 |
| **Resting time** | | | | | |
| No predator Strong - Predator cue Strong | -28.19 | 13.0 | 237 | -2.171 | 0.1344 |
| No predator Strong - No predator Weak | -30.64 | 10.6 | 237 | -2.883 | **0.0222** |
| No predator Strong - Predator cue Weak | -4.49 | 11.0 | 237 | -0.408 | 0.9770 |
| Predator cue Strong - No predator Weak | -2.45 | 12.9 | 237 | -0.190 | 0.9976 |
| Predator cue Strong - Predator cue Weak | 23.70 | 13.2 | 237 | 1.792 | 0.2799 |
| No predator weak - Predator cue Weak | 26.15 | 10.9 | 237 | 2.394 | 0.0809 |
| **Feeding rate** | | | | | |
| Weak Predator Yes - Strong Predator Yes | -2.501 | 0.908 | 288 | -2.753 | **0.0317** |
| Weak Predator Yes - Weak Predator No | 1.839 | 0.903 | 288 | 2.037 | 0.1770 |
| Weak Predator Yes - Strong Predator No | -0.662 | 1.260 | 288 | -0.524 | 0.9532 |
| Strong Predator Yes - Weak Predator No | 4.339 | 1.300 | 288 | 3.341 | **0.0052** |
| Strong Predator Yes - Strong Predator No | 1.839 | 0.903 | 288 | 2.037 | 0.1770 |
| Weak Predator No - Strong Predator No | -2.501 | 0.908 | 288 | -2.753 | **0.0317** |
| **MDA** | | | | | |
| Weak Metal Yes Predator Yes - Weak Metal No Predator Yes | 0.2710 | 0.0576 | 325 | 4.702 | **0.0001** |
| Weak Metal Yes Predator Yes - Strong Metal No Predator Yes | 0.4616 | 0.0638 | 325 | 7.231 | **<.0001** |
| Weak Metal Yes Predator Yes - Weak Metal Yes Predator No | -0.0761 | 0.0440 | 325 | -1.731 | 0.6670 |
| Weak Metal Yes Predator Yes - Strong Metal Yes Predator No | 0.3887 | 0.0753 | 325 | 5.162 | **<.0001** |
| Weak Metal Yes Predator Yes - Weak Metal No Predator No | 0.1949 | 0.0713 | 325 | 2.734 | 0.1164 |
| Weak Metal Yes Predator Yes - Strong Metal No Predator No | 0.3855 | 0.0779 | 325 | 4.949 | **<.0001** |
| Strong Metal Yes Predator Yes - Weak Metal No Predator Yes | -0.1938 | 0.0623 | 325 | -3.111 | **0.0420** |
| Strong Metal Yes Predator Yes - Strong Metal No Predator Yes | -0.0032 | 0.0681 | 325 | -0.047 | 1.0000 |
| Strong Metal Yes Predator Yes - Weak Metal Yes Predator No | -0.5409 | 0.0753 | 325 | -7.183 | **<.0001** |
| Strong Metal Yes Predator Yes - Strong Metal Yes Predator No | -0.0761 | 0.0440 | 325 | -1.731 | 0.6670 |
| Strong Metal Yes Predator Yes - Weak Metal No Predator No | -0.2699 | 0.0751 | 325 | -3.592 | **0.0090** |
| Strong Metal Yes Predator Yes - Strong Metal No Predator No | -0.0793 | 0.0814 | 325 | -0.974 | 0.9778 |
| Weak Metal No Predator Yes - Strong Metal No Predator Yes | 0.1906 | 0.0650 | 325 | 2.934 | 0.0693 |
| Weak Metal No Predator Yes - Weak Metal Yes Predator No | -0.3471 | 0.0736 | 325 | -4.714 | **0.0001** |
| Weak Metal No Predator Yes - Strong Metal Yes Predator No | 0.1177 | 0.0773 | 325 | 1.522 | 0.7952 |
| Weak Metal No Predator Yes - Weak Metal No Predator No | -0.0761 | 0.0440 | 325 | -1.731 | 0.6670 |
| Weak Metal No Predator Yes - Strong Metal No Predator No | 0.1145 | 0.0799 | 325 | 1.434 | 0.8411 |
| Strong Metal No Predator Yes - Weak Metal Yes Predator No | -0.5377 | 0.0771 | 325 | -6.972 | **<.0001** |
| Strong Metal No Predator Yes - Strong Metal Yes Predator No | -0.0729 | 0.0807 | 325 | -0.904 | 0.9856 |
| Strong Metal No Predator Yes - Weak Metal No Predator No | -0.2667 | 0.0770 | 325 | -3.465 | **0.0138** |
| Strong Metal No Predator Yes - Strong Metal No Predator No | -0.0761 | 0.0440 | 325 | -1.731 | 0.6670 |
| Weak Metal Yes Predator No - Strong Metal Yes Predator No | 0.4648 | 0.0611 | 325 | 7.601 | **<.0001** |
| Weak Metal Yes Predator No - Weak Metal No Predator No | 0.2710 | 0.0576 | 325 | 4.702 | **0.0001** |
| Weak Metal Yes Predator No - Strong Metal No Predator No | 0.4616 | 0.0638 | 325 | 7.231 | **<.0001** |
| Strong Metal Yes Predator No - Weak Metal No Predator No | -0.1938 | 0.0623 | 325 | -3.111 | **0.0420** |
| Strong Metal Yes Predator No - Strong Metal No Predator No | -0.0032 | 0.0681 | 325 | -0.047 | 1.0000 |
| Weak Metal No Predator No - Strong Metal No Predator No | 0.1906 | 0.0650 | 325 | 2.934 | 0.0693 |
| **CEA** | | | | | |
| No predator Weak Predator No - Predator cue Weak Predator No | -0.1318 | 0.0365 | 322 | -3.615 | **0.0083** |
| No predator Weak Predator No - No predator Strong Predator No | -0.1552 | 0.0461 | 322 | -3.365 | **0.0192** |
| No predator Weak Predator No - Predator cue Strong Predator No | -0.0106 | 0.0559 | 322 | -0.190 | 1.0000 |
| No predator Weak Predator No - No predator Weak Predator Yes | -0.0755 | 0.0365 | 322 | -2.069 | 0.4373 |
| No predator Weak Predator No - Predator cue Weak Predator Yes | -0.2073 | 0.0514 | 322 | -4.033 | **0.0018** |
| No predator Weak Predator No - No predator Strong Predator Yes | -0.1210 | 0.0378 | 322 | -2.624 | 0.1512 |
| No predator Weak Predator No - Predator cue Strong Predator Yes | 0.0236 | 0.0555 | 322 | 0.425 | 0.9999 |
| Predator cue Weak Predator No - No predator Strong Predator No | -0.0235 | 0.0457 | 322 | -0.513 | 0.9996 |
| Predator cue Weak Predator No - Predator cue Strong Predator No | 0.1212 | 0.0556 | 322 | 2.180 | 0.3666 |
| Predator cue Weak Predator No - No predator Weak Predator Yes | 0.0563 | 0.0517 | 322 | 1.088 | 0.9589 |
| Predator cue Weak Predator No - Predator cue Weak Predator Yes | -0.0755 | 0.0365 | 322 | -2.069 | 0.4373 |
| Predator cue Weak Predator No - No predator Strong Predator Yes | 0.0107 | 0.0457 | 322 | 0.235 | 1.0000 |
| Predator cue Weak Predator No - Predator cue Strong Predator Yes | 0.1554 | 0.0551 | 322 | 2.818 | 0.0942 |
| No predator Strong Predator No - Predator cue Strong Predator No | 0.1446 | 0.0481 | 322 | 3.009 | 0.0563 |
| No predator Strong Predator No - No predator Weak Predator Yes | 0.0798 | 0.0469 | 322 | 1.701 | 0.6867 |
| No predator Strong Predator No - Predator cue Weak Predator Yes | -0.0520 | 0.0463 | 322 | -1.124 | 0.9512 |
| No predator Strong Predator No - No predator Strong Predator Yes | 0.0342 | 0.0436 | 322 | 0.785 | 0.9938 |
| No predator Strong Predator No - Predator cue Strong Predator Yes | 0.1788 | 0.0645 | 322 | 2.773 | 0.1056 |
| Predator cue Strong Predator No - No predator Weak Predator Yes | -0.0648 | 0.0566 | 322 | -1.146 | 0.9459 |
| Predator cue Strong Predator No - Predator cue Weak Predator Yes | -0.1966 | 0.0561 | 322 | -3.508 | **0.0120** |
| Predator cue Strong Predator No - No predator Strong Predator Yes | -0.1104 | 0.0652 | 322 | -1.692 | 0.6923 |
| Predator cue Strong Predator No - Predator cue Strong Predator Yes | 0.0342 | 0.0436 | 322 | 0.785 | 0.9938 |
| No predator Weak Predator Yes - Predator cue Weak Predator Yes | -0.1318 | 0.0365 | 322 | -3.615 | **0.0083** |
| No predator Weak Predator Yes - No predator Strong Predator Yes | -0.0456 | 0.0469 | 322 | -0.972 | 0.9781 |
| No predator Weak Predator Yes - Predator cue Strong Predator Yes | 0.0990 | 0.0561 | 322 | 1.764 | 0.6446 |
| Predator cue Weak Predator Yes - No predator Strong Predator Yes | 0.0862 | 0.0463 | 322 | 1.863 | 0.5775 |
| Predator cue Weak Predator Yes - Predator cue Strong Predator Yes | 0.2308 | 0.0556 | 322 | 4.151 | **0.0011** |
| No predator Strong Predator Yes - Predator cue Strong Predator Yes | 0.1446 | 0.0481 | 322 | 3.009 | 0.0563 |


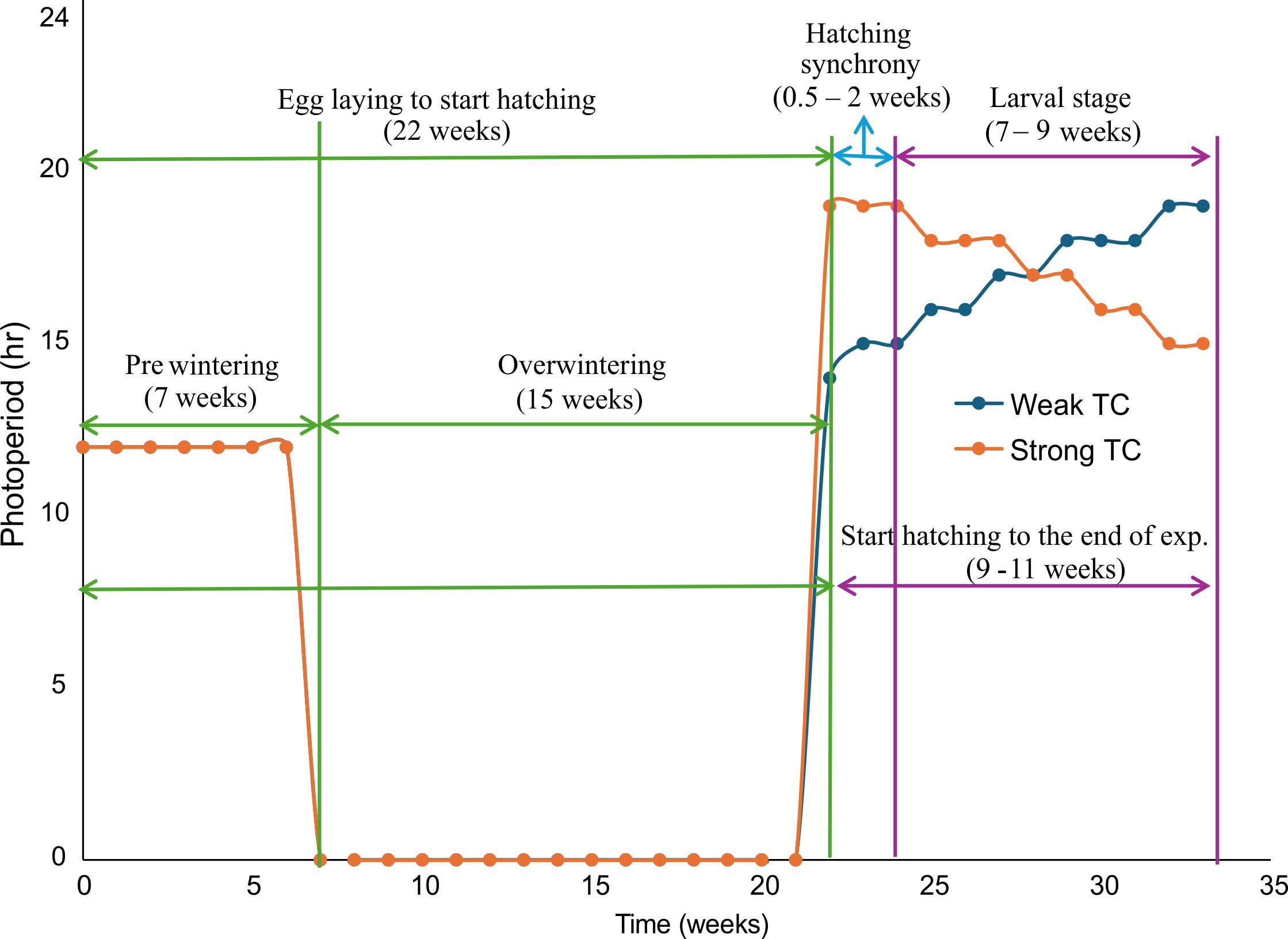


## Figure S1. Photoperiod regimes of weak and strong time constraint groups used during the experiment. The x-axis represents the timeline of the experiment in weeks, from egg hatching (week 0) until the last larva finished 5-days treatments after entering F-0 instar (week 36).

## Figure S2. Average daily temperature from 1 May 2023 to 14 July 2023 in pond which are located in in NW Poland (53°39'27.6"N, 16°16'26.6"E).

##
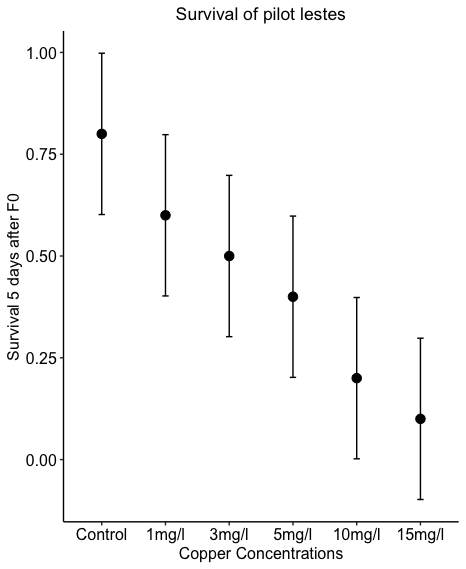


## Figure S3. Pilot toxicity study. Effect of different copper concentrations (0, 1, 3, 5, 10, and 15 mg/l) on the survival of *L. sponsa* larvae during larval treatment (survival 5 days after F-0). Error bars show 95% CI.
